# An angiopoietin 2, FGF23, and BMP10 biomarker signature differentiates atrial fibrillation from other concomitant cardiovascular conditions

**DOI:** 10.1038/s41598-023-42331-7

**Published:** 2023-10-05

**Authors:** Winnie Chua, Victor R. Cardoso, Eduard Guasch, Moritz F. Sinner, Christoph Al-Taie, Paul Brady, Barbara Casadei, Harry J. G. M. Crijns, Elton A. M. P. Dudink, Stéphane N. Hatem, Stefan Kääb, Peter Kastner, Lluis Mont, Frantisek Nehaj, Yanish Purmah, Jasmeet S. Reyat, Ulrich Schotten, Laura C. Sommerfeld, Stef Zeemering, André Ziegler, Georgios V. Gkoutos, Paulus Kirchhof, Larissa Fabritz

**Affiliations:** 1 Institute of Cardiovascular Sciences, University of Birmingham, Birmingham, UK; 2 MRC Health Data Research UK (HDR), Midlands Site, London, UK; 3 Institute of Cancer and Genomic Sciences, University of Birmingham, Birmingham, UK; 4 Hospital Clinic de Barcelona, Institute of Biomedical Research August Pi Sunyer (IDIBAPS), Barcelona, Spain; 5 Department of Medicine I, University Hospital, LMU, Munich, Germany; 6 German Centre for Cardiovascular Research (DZHK), Partner Site: Munich Heart Alliance, Munich, Germany; 7 University Center of Cardiovascular Science, University Heart and Vascular Center Hamburg, University Medical Center Hamburg-Eppendorf, UKE Martinistrasse 52, 20246 Hamburg, Germany; 8 German Centre for Cardiovascular Research (DZHK), Partner Site: Hamburg/Kiel/Lübeck, Hamburg, Germany; 9 Department of Cardiology, University Heart and Vascular Center Hamburg, University Medical Center Hamburg-Eppendorf, Hamburg, Germany; 10 Sandwell and West Birmingham Hospitals NHS Trust, Birmingham, UK; 11 University of Oxford, Oxford, UK; 12 Cardiovascular Research Institute Maastricht (CARIM), Maastricht University, Maastricht, The Netherlands; 13 IHU-ICAN Institute of Cardiometabolism and Nutrition, Paris, France; 14 Roche Diagnostics GmbH, Penzberg, Germany; 15 Roche Diagnostics International AG, Rotkreuz, Switzerland

**Keywords:** Biomarkers, Cardiology

## Abstract

Early detection of atrial fibrillation (AF) enables initiation of anticoagulation and early rhythm control therapy to reduce stroke, cardiovascular death, and heart failure. In a cross-sectional, observational study, we aimed to identify a combination of circulating biomolecules reflecting different biological processes to detect prevalent AF in patients with cardiovascular conditions presenting to hospital. Twelve biomarkers identified by reviewing literature and patents were quantified on a high-precision, high-throughput platform in 1485 consecutive patients with cardiovascular conditions (median age 69 years [Q1, Q3 60, 78]; 60% male). Patients had either known AF (45%) or AF ruled out by 7-day ECG-monitoring. Logistic regression with backward elimination and a neural network approach considering 7 key clinical characteristics and 12 biomarker concentrations were applied to a randomly sampled discovery cohort (n = 933) and validated in the remaining patients (n = 552). In addition to age, sex, and body mass index (BMI), BMP10, ANGPT2, and FGF23 identified patients with prevalent AF (AUC 0.743 [95% CI 0.712, 0.775]). These circulating biomolecules represent distinct pathways associated with atrial cardiomyopathy and AF. Neural networks identified the same variables as the regression-based approach. The validation using regression yielded an AUC of 0.719 (95% CI 0.677, 0.762), corroborated using deep neural networks (AUC 0.784 [95% CI 0.745, 0.822]). Age, sex, BMI and three circulating biomolecules (BMP10, ANGPT2, FGF23) are associated with prevalent AF in unselected patients presenting to hospital. Findings should be externally validated. Results suggest that age and different disease processes approximated by these three biomolecules contribute to AF in patients. Our findings have the potential to improve screening programs for AF after external validation.

## Introduction

Cardioembolic stroke is a common complication of atrial fibrillation (AF) effectively prevented by oral anticoagulation^1–3^. Treatment of concomitant cardiovascular conditions, and early rhythm control therapy can further improve outcomes in people with AF^4^. Early detection of AF is therefore a recognised priority for cardiovascular health^1,5^. Simple methods to identify people at high risk of AF who would benefit from early AF detection are needed to implement timely care and prevent AF complications. While point-of-care testing for AF, using ECG recordings, is being tested in unselected elderly populations^6^, and in view of consumer-electronics-based methods that can provide access to rhythm screening at scale^7,8^, there is a clear need to target screening efforts to at-risk populations to contain the effort and resources required.

Biomarkers can render ECG screening more effective and efficient^5,9^. Quantification of the circulating biomolecule N-terminal pro B-type natriuretic peptide (NTproBNP) was shown to enable targeted screening, leading to lower stroke rates during long-term follow-up compared to usual care^9^. However, NTproBNP is also elevated in other cardiovascular conditions, notably in heart failure. So far, there is no single “best” biomarker for AF. In addition to NTproBNP^9,10^, C-reactive protein^11^, cardiac troponins, and fibroblast growth factor 23 (FGF23)^12–14^ hold promise. As multiple pathophysiological pathways are implicated by the different mechanisms that lead to AF^15,16^, combining several biomarkers reflecting these processes could improve detection of people with AF.

In this study, we quantified known and novel biomarkers utilising a high-precision, high-throughput analytical platform in a cohort of multi-ethnic patients with cardiovascular conditions. Biomolecules that are relevant for AF and represent different disease processes were selected in a modified Delphi-process as part of the CATCH ME consortium^15^. All patients without known AF underwent 7-day Holter monitoring to rule out undetected AF^17^. We applied both statistical modelling and machine learning techniques to identify biomarkers that enhance detection of unknown prevalent AF.

## Methods

### Selection of candidate biomarkers

Biomolecules were selected based on a modified Delphi process. A thorough literature and patent search was performed for biomarkers of AF in 2018. To assess the existing knowledge on biomarkers, we searched PubMed, the Cochrane Library, Scopus, and databases of the European Patent Office (EPO) and United States Patent and Trademark Office (USPTO) with no language or date restrictions to identify research and patents describing biomarkers related to AF. Search terms, including various alternate spellings, included “atrial fibrillation”, “screening”, “blood”, “plasma” and “biomarkers”.

An expert consensus process integrating knowledge on biomarkers, AF mechanisms, and AF screening, was coordinated to identify promising biomarkers for AF reflecting different disease processes in several in-person and remote meetings. This process was designed along the principles of a Delphi process and relied on face-to-face discussion and agreement during meetings in addition to online surveys.

Integrating expert knowledge with the literature and patent review, an iterative collaborative discussion amongst experts within the CATCH ME Consortium (www.catch-me.info) identified 12 candidate biomarkers for AF, namely: angiopoietin 2 (ANGPT2), bone morphogenetic protein 10 (BMP10), cancer antigen 125 (CA125), C-reactive protein (CRP), endothelial cell specific molecule 1 (ESM1), fatty acid binding protein 3 (FABP3), FGF23, growth differentiation factor 15 (GDF15), insulin-like growth factor binding protein 7 (IGFBP7), interleukin 6 (IL6), NTproBNP, and troponin T (TnT).

The 12 biomarkers selected were quantified and taken forward for testing in the present study. Seven clinical characteristics (age, sex, body mass index (BMI), estimated glomerular filtration rate (eGFR), heart failure, stroke/ transient ischemic attack (TIA), hypertension) were selected based on a separate literature review and on-going analysis of clinical predictors for AF^18^.

### Study population

Consecutive patients, referred to the Sandwell and West Birmingham NHS Trust (Birmingham, UK) for inpatient or outpatient evaluation of acute illnesses, were recruited between September 2014 and February 2018 to the Birmingham and Black Country Atrial Fibrillation Registry (BBC-AF). Patients had either diagnosed AF confirmed by ECG or presented with at concomitant cardiovascular conditions as assessed by the CHA_2_DS_2_VASc score with either one of the following: age greater than 75 years or stroke, or two of the following: age greater than 65 years, female sex, congestive heart failure, hypertension, diabetes, prior stroke, or transischemic attack, left ventricular hypertrophy or vascular disease). Details of the enrolment criteria have been published previously^12^. Patients who did not have a diagnosis of AF underwent 7-day ambulatory ECG monitoring to rule out asymptomatic AF. There were very few exclusion criteria (age < 18 years, inability to consent, to follow up and unwillingness to undergo investigations or life expectancy < 1 year). The patients were consecutively enrolled in both hospital outpatient clinics and inpatient admission settings with the majority being admitted as inpatients.

### Ethics declaration

This study complied with the Declaration of Helsinki, was approved by the National Research Ethics Service Committee (IRAS ID 97753) and was sponsored by the University of Birmingham. All patients provided written informed consent.

### Biomarker quantification

Blood samples from all patients were spun, fractionated, frozen, and stored at − 80 °C until analysis. Absolute protein concentrations were centrally quantified in EDTA plasma (see Supplemental material S1 for details). Run controls and calibrators were measured twice each run, and staff involved were blinded to clinical status and data.

### Analysis

Using random selection, the cohort was divided at a 60:40 ratio, conventional for discovery-validation paradigms in regression modelling and machine learning. For the identification of biomarkers in the discovery cohort, we considered 12 biomarkers and 7 clinical risk factors (hypertension, heart failure, history of stroke or transient ischaemic attack (TIA), kidney function, and body mass index (BMI); age and sex were fixed factors). Hypertension was defined as an elevated resting blood pressure or hypertension requiring anti-hypertensive therapy. Heart failure was defined as left ventricular ejection fraction of < 50% or moderate or severe left ventricular dysfunction as an established diagnosis, or a clinical diagnosis of heart failure with a New York Heart Association (NYHA) Functional Classification class III or IV. Kidney function was determined based on the estimated glomerular filtration rate (eGFR) using centrally quantified creatinine levels, calculated using the (CKD-EPI) equation^19^. Since there were minimal missing data, only complete cases were used for analysis (Supplemental material Fig. S1).

Baseline characteristics of patients with and without AF were compared in the discovery and validation cohorts using Chi^2^ tests, independent samples t-tests, or Mann–Whitney U tests as applicable after checking for data normality (Kolmogorov–Smirnov test and visual inspection of descriptive plots). A two-tailed P value of < 0.05 was considered statistically significant. Unadjusted univariate analyses indicated the relationship of each variable to the study’s outcome (rhythm status: AF or No AF). Biomarkers were also adjusted to account for common confounders. We evaluated the variance inflation factor to identify possible collinearity.

A logistic regression with backward elimination was applied to the discovery dataset (n = 933; 44% with AF) to select variables using a p value of 0.157 which is a recommended proxy for the Akaike information criterion (AIC)^20^. The selected variables from this process (apparent model) with increased odds of indicating AF (odds ratio, OR > 1), were bootstrapped to account for potential overfitting as a means of internal validation. The variables were subsequently validated using data from the validation cohort by fitting the bootstrapped coefficients to the data. The performance of the apparent, bootstrapped, and validation models were assessed by calculating the area under the ROC curve (AUC) and 95% confidence intervals (CI). For each model, the sensitivity, specificity, positive predictive value, and negative predictive value were also calculated. We applied different cutoff values to evaluate model performance in discriminating between patients at low and high risk of prevalent AF.

To assess the robustness of our findings, we evaluated the effect of replacing the biomarkers in our model with the current ‘industry standard’ biomarker NTproBNP^9^. We also compared the performance of our model with the CHARGE-AF score and STROKESTOPII criteria by fitting available data of our whole cohort using the coefficients and criteria for those studies.

### Machine learning

To complement statistical analyses and assess an alternative approach for interrogating the dataset, machine learning models were developed and applied on training and test datasets, corresponding to the discovery and validation cohorts of the regression analysis, with an additional internal-validation set created using 20% of the training data. We employed a Neural Network algorithm using the Keras open source library. Data were pre-processed using the Scikit-learn software. Categorical variables (sex, heart failure, hypertension, stroke/TIA) were transformed using min–max scaler. Continuous variables were scaled towards mean and scaled to unit variance in reference to the training set. The model contained 2 blocks with a layer of 256 hidden dense variables with RELU activation, followed by a dropout layer. Subsequently, a 1 node dense layer with sigmoid activation was used for the prediction. The model was trained using the adam optimiser until the model’s performance plateaued for 20 epochs. The best performing model was selected using binary cross-entropy. 10 further models, using different starting variables, were trained and evaluated using the Shapley Additive exPlanations (SHAP) method to identify the influence of individual variables in the neural network by quantifying feature importance for a linear model in the presence of multicollinearity. SHAP is the current state-of-the-art procedure for interpretation of neural networks. To increase robustness, the neural network algorithm was run with 100 different initialisation seeds and the importance values with their corresponding confidence intervals were assessed. References to machine learning algorithms are in Supplemental material S1.

In Supplemental Methods S1, we report the outcomes of all models with biomarkers which are rank normalised by Blom transformation for better comparability between biomarkers.

All analyses were completed using SPSS v. 24 (IBM Corporation, Armonk, NY, USA) and R programming language^21^.

## Results

### Patient characteristics

1485 patients (36% female, mean age 69 years, 45% with diagnosed AF) were included in the analysis (Supplemental Material Fig. S1). As expected, patients with AF were older, and more often had a history of heart failure. History of stroke / transient ischemic attack (TIA) and body mass index did neither differed between patients with or without AF or between in- and outpatient groups (Table 1, Supplemental Material Tables S4, S12). 16 new AF cases were identified by the 7-day Holter and were included in the AF group. All patterns of AF, paroxysmal and non-paroxysmal, were included.

**Table 1:** Clinical characteristics of the randomly selected discovery cohort.

Characteristic	No AF	AF	P-value	Univariable analysis
	N = 522	N = 411		Odds ratio (95% CI)
Age, years*	67 (58, 75)	74 (66, 80)	< 0.001	1.043 (1.031, 1.055)
Sex, males	309 (59%)	256 (62%)	0.337	1.138 (0.873, 1.484)
Ethnicity				
Caucasian	362 (69%)	350 (85%)	< 0.001	Reference
Asian	112 (22%)	31 (8%)		0.286 (0.187, 0.437)
Afro-Caribbean	48 (9%)	30 (7%)		0.646 (0.400, 1.044)
BMI, kg/m^2^*	28.7 (25.5, 32.5)	29.0 (25.1, 33.1)	0.627	1.008 (0.987, 1.029)
eGFR, mL/min/1.73 m^2^	71.7 (26.1)	67.8 (25.9)	0.023	0.994 (0.989, 0.999)
Diabetes	238 (46%)	96 (23%)	< 0.001	0.364 (0.273, 0.484)
Stroke/TIA	46 (9%)	38 (9%)	0.818	1.054 (0.672, 1.654)
Coronary artery disease	252 (48%)	93 (23%)	< 0.001	0.313 (0.235, 0.418)
Hypertension	333 (64%)	218 (53%)	0.001	0.641 (0.493, 0.834)
Heart failure	222 (43%)	219 (53%)	0.001	1.541 (1.188, 1.999)
Admission criteria (inpatient)	469 (90%)	293 (71%)	< 0.001	0.281 (0.197, 0.400)
Medication				
NOAC	12 (2%)	198 (48%)	< 0.001	39.507 (21.590, 72.291)
VKA	12 (2%)	100 (24%)	< 0.001	13.666 (7.387, 25.281)
Aspirin	362 (69%)	101 (25%)	< 0.001	0.144 (0.108, 0.193)
Antiplatelet agents	277 (53%)	76 (19%)	< 0.001	0.201 (0.148, 0.272)
ACE inhibitors	194 (37%)	121 (29%)	0.013	0.705 (0.535, 0.930)
Angiotensin II receptor blocker	82 (16%)	68 (17%)	0.730	1.064 (0.749, 1.511)
Beta-blocker	306 (59%)	227 (55%)	0.299	0.871 (0.671, 1.131)
Diuretic	164 (31%)	172 (42%)	0.001	1.571 (1.200, 2.057)
Calcium channel antagonist	116 (22%)	66 (16%)	0.018	0.670 (0.479, 0.936)
Cardiac glycoside	3 (1%)	89 (22%)	< 0.001	47.817 (15.007, 152.360)
Aldosterone antagonist	37 (7%)	33 (8%)	0.588	1.144 (0.702, 1.865)
Antiarrhythmics	8 (2%)	37 (9%)	< 0.001	6.356 (2.926, 13.807)
Biomarkers				
ANGPT2 (ng/mL)*	2.36 (1.73, 3.45)	3.64 (2.28, 6.14)	< 0.001	1.243 (1.177, 1.313)
BMP10 (ng/mL)*	1.95 (1.70, 2.32)	2.35 (1.94, 2.94)	< 0.001	2.953 (2.348, 3.715)
CRP (mg/L)*	4.95 (1.63, 18.89)	4.19 (1.57, 15.59)	0.386	0.999 (0.996, 1.001)
CA125 (per 10 U/mL)*	1.23 (0.82, 2.01)	1.57 (0.95, 3.40)	< 0.001	1.050 (1.020, 1.081)
ESM1 (ng/mL)*	2.01 (1.47, 2.91)	2.36 (1.78, 3.43)	< 0.001	1.137 (1.062, 1.218)
FGF23 (per 100 pg/mL)*	1.65 (1.05, 2.69)	1.97 (1.35, 4.16)	< 0.001	1.050 (1.023, 1.077)
FABP3 (per 10 ng/mL)*	3.53 (2.63, 5.19)	3.77 (2.82, 5.92)	0.017	1.001 (0.986, 1.016)
GDF15 (per 100 pg/mL)*	18.71 (11.42, 31.08)	21.29 (13.41, 35.22)	0.004	1.004 (1.000, 1.009)
IGFBP7 (ng/mL)*	96.23 (82.74, 115.30)	110.17 (91.65, 140.09)	< 0.001	1.010 (1.006, 1.013)
IL6 (pg/mL)*	6.38 (3.31, 14.66)	6.49 (3.37, 14.69)	0.691	1.001 (0.995, 1.008)
NTproBNP (per 100 pg/mL)*	4.21 (1.08, 14.34)	11.20 (3.51, 28.61)	< 0.001	1.006 (1.002, 1.009)
TnT (per 100 pg/mL)*	0.30 (0.12, 1.09)	0.22 (0.12, 0.50)	0.001	0.963 (0.943, 0.984)

Categorical variables are reported as n (%), continuous variables are reported as mean (standard deviation) or median (quartile 1, quartile 3) for skewed distributions (*). The independent t-test (or Mann–Whitney U test for skewed distributions) and Χ2 tests were used to compare characteristics between patients. eGFR was estimated using the CKD-EPI equation.

*BMI* body mass index, *eGFR* estimated glomerular filtration rate, *TIA* transient ischemic attack, *NOAC* non-vitamin K antagonist oral anticoagulant, *VKA* vitamin K antagonist, *ACE* angiotensin-converting enzyme, *ANGPT2* angiopoietin 2, *BMP10* bone morphogenetic protein 10, *CRP* high-sensitivity C-reactive protein, *CA125* cancer antigen 125, *ESM1* endothelial cell specific molecule 1, *FGF23* fibroblast growth factor 23, *FABP3* fatty acid binding protein 3, *GDF15* growth differentiation factor 15, *IGFBP7* insulin like growth factor binding protein 7, *IL6* interleukin 6, *NTproBNP* N-terminal pro-B-type natriuretic peptide, *TnT* high-sensitivity cardiac troponin T.

### Elevated ANGPT2, BMP10, FGF23, IGFBP7, and NTproBNP are associated with increased risk of AF

**Figure 1:**
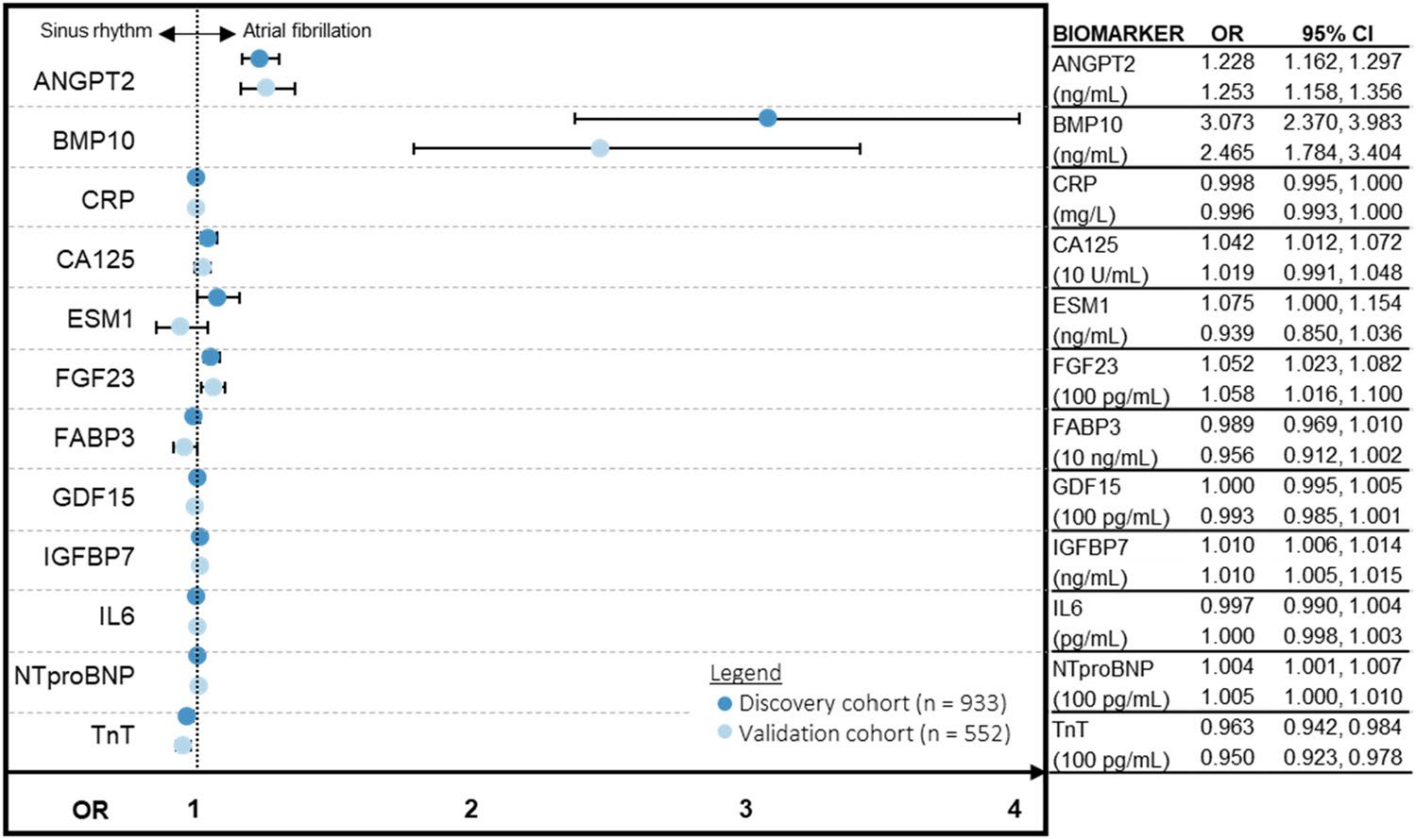
Five biomarkers predict prevalent AF. Higher levels of ANGPT2, BMP10, FGF23, IGFBP7, and NTproBNP are consistently associated with increased odds of prevalent AF as suggested by univariate OR and 95% CI of quantified biomarkers for the discovery and validation cohorts. Biomarker levels have been adjusted for age, sex, BMI, eGFR, heart failure, stroke/TIA, and hypertension status. *BMI* body mass index, *eGFR* estimated glomerular filtration rate, *TIA* transient ischemic attack, *ANGPT2* angiopoietin 2, *BMI* body mass index, *BMP10* bone morphogenetic protein 10, *CRP* high-sensitivity C-reactive protein, *CA125* cancer antigen 125, *CI* confidence intervals, *eGFR* estimated glomerular filtration rate, *ESM1* endothelial cell specific molecule 1, *FGF23* fibroblast growth factor 23, *FABP3* fatty acid binding protein 3, *GDF15* growth differentiation factor 15, *IGFBP7* insulin like growth factor binding protein 7, *IL6* interleukin 6, *NTproBNP* N-terminal pro-B-type natriuretic peptide, *OR* odds ratio, *TnT* high-sensitivity cardiac troponin T.

We interrogated the univariate association of biomarkers to the outcome of AF, adjusted by established clinical risk factors (age, sex, BMI, eGFR, heart failure, stroke/TIA, hypertension) (Fig. 1). Five associations were consistent between the discovery and validation cohorts—elevated levels of ANGPT2, BMP10, FGF23, IGFBP7, and NTproBNP significantly increased odds of AF. Conversely, CA125 and ESM1 were associated with increased odds of AF in the discovery cohort but this was not confirmed in the validation cohort. CRP, FABP3, GDF15, IL6 were not associated with the outcome whereas lower TnT concentrations were associated with AF in this cohort attending in- or outpatient care at the hospital.

### Optimism-adjusted model

**Figure 2:**
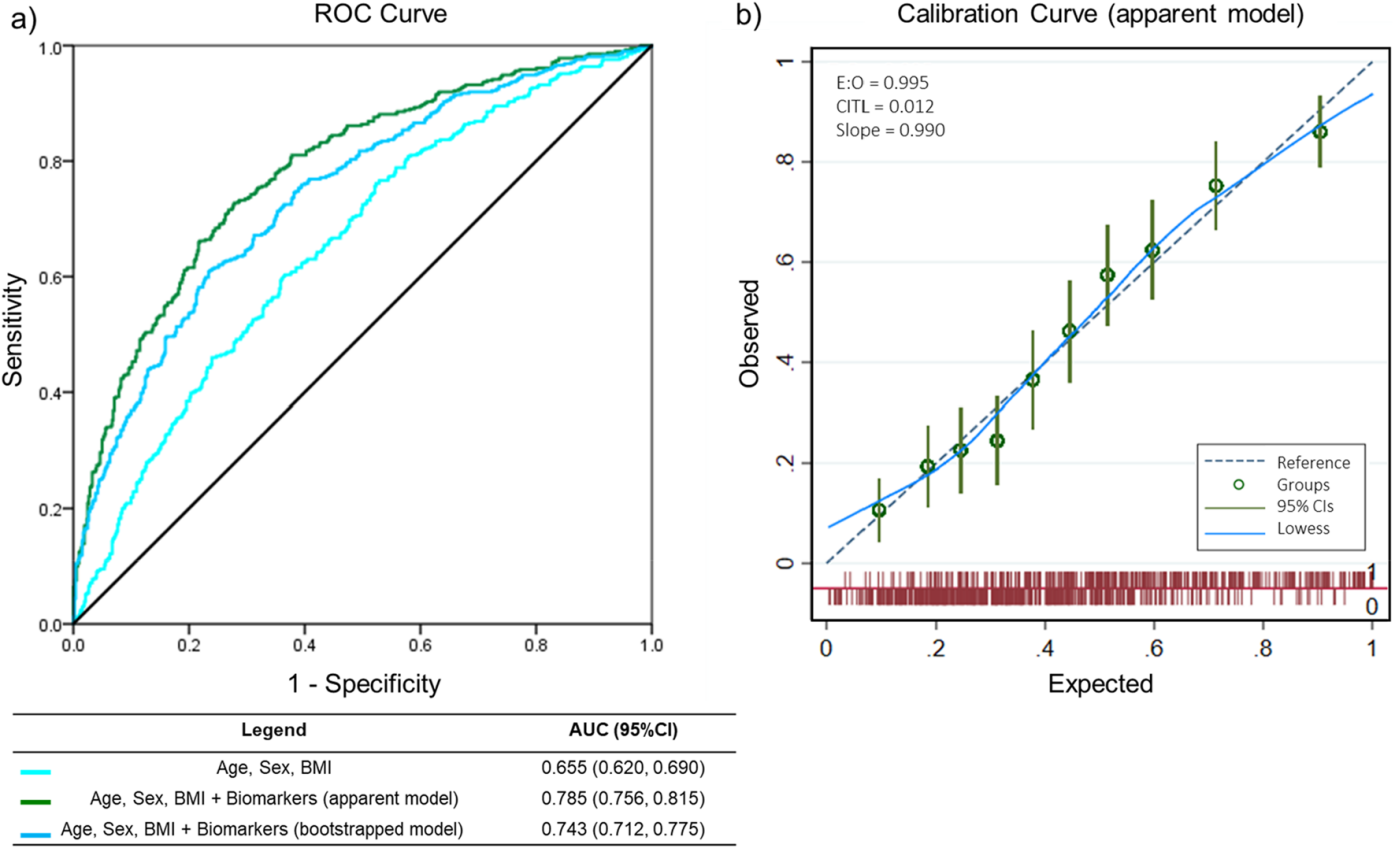
Biomarkers improve prediction of prevalent AF. (**a**) ANGPT2, BMP10, and FGF23 provided added value to clinical variables (age, sex, BMI) as denoted by the increase of AUC from 0.655 (95% CI 0.620, 0.690) to 0.785 (95% CI 0.756, 0.815) for the apparent model; [AUC 0.743 (95% CI 0.712, 0.775) after optimism adjustment using bootstrapping]. (**b**) Calibration curve of the apparent model demonstrating near perfect calibration as expected for model development. *ANGPT2* angiopoietin 2, *AUC* area under the ROC curve, *BMP10* bone morphogenetic protein 10, *CI* confidence intervals, *FGF23* fibroblast growth factor 23.

The backward elimination process resulted in an apparent model that included 4 clinical characteristics and 6 biomarkers (Supplemental Material Table S1). As we were interested in variables whose increase indicated greater odds of having AF, we fitted and bootstrapped an optimism adjusted model using variables with a significant OR of > 1 (Supplemental Material Table S2). The AUC for the apparent model was 0.784 (95% CI 0.756, 0.815), sensitivity 60% and specificity 81%. The optimism-adjusted model had a marginal shrinkage of all these measurements, resulting in an AUC of 0.743 (95% CI 0.712, 0.775; Fig. 2), sensitivity 52% and specificity 80% (Table 2). To estimate the added value of each individual biomarker to the clinical characteristics (age, sex, BMI), we calculated the AUC for each biomarker and ranked them by change in AUC (Supplemental Material Table S3).

**Table 2:** Combination of age, sex, BMI, ANGPT2, BMP10, FGF23 for prediction of prevalent AF.

Model	C-statistic (95% CI)	Sensitivity (%, 95% CI)	Specificity (%, 95% CI)	Positive predictive value (%, 95% CI)	Negative predictive value (%, 95% CI)
Apparent	0.785 (0.756, 0.815)	59.85 (54.94, 64.63)	81.21 (77.61, 84.46)	71.30 (67.17, 75.11)	72.18 (69.60, 74.61)
Optimism-adjusted	0.743 (0.712, 0.775)	51.83 (47.03, 56.61)	79.57 (75.98, 82.84)	66.47 (62.18, 70.50)	67.89 (65.54, 70.16)
Validation	0.719 (0.677, 0.762)	51.05 (45.10, 56.98)	81.49 (76.70, 85.67)	71.92 (66.38, 76.87)	64.19 (61.16, 67.12)

Performance measures of the apparent, optimism adjusted and validation models.

*AF* atrial fibrillation, *BMI* body mass index, *ANGPT2* angiopoietin 2, *BMP10* bone morphogenetic protein 10, *FGF23* fibroblast growth factor 23.

### Model validation

In the validation cohort (n = 552; Supplemental Material Table S4), patients with AF had a similar clinical and biomarker profile compared to the discovery cohort. The coefficients from the optimism-adjusted model were fitted with these data. The model performed consistently with an AUC of 0.719 (95% CI 0.677, 0.762), sensitivity 51%, and specificity 81%.

The ROC curve considers all consecutive cutoff points to define a high risk and a low risk group. The performance of the model was evaluated at cutoff points of 10%, 20%, 30%, and 40% probability of being classified as “at risk”. For each cutoff point, we illustrated the classifications generated by the model in identifying patients whom the model considers “at risk”, “not at risk”, and estimated the number of patients who would be correctly identified as well as those potentially missed (Supplemental Material Table S5).

### Neural network analysis confirms ANGPT2, BMP10, FGF23

**Figure 3:**
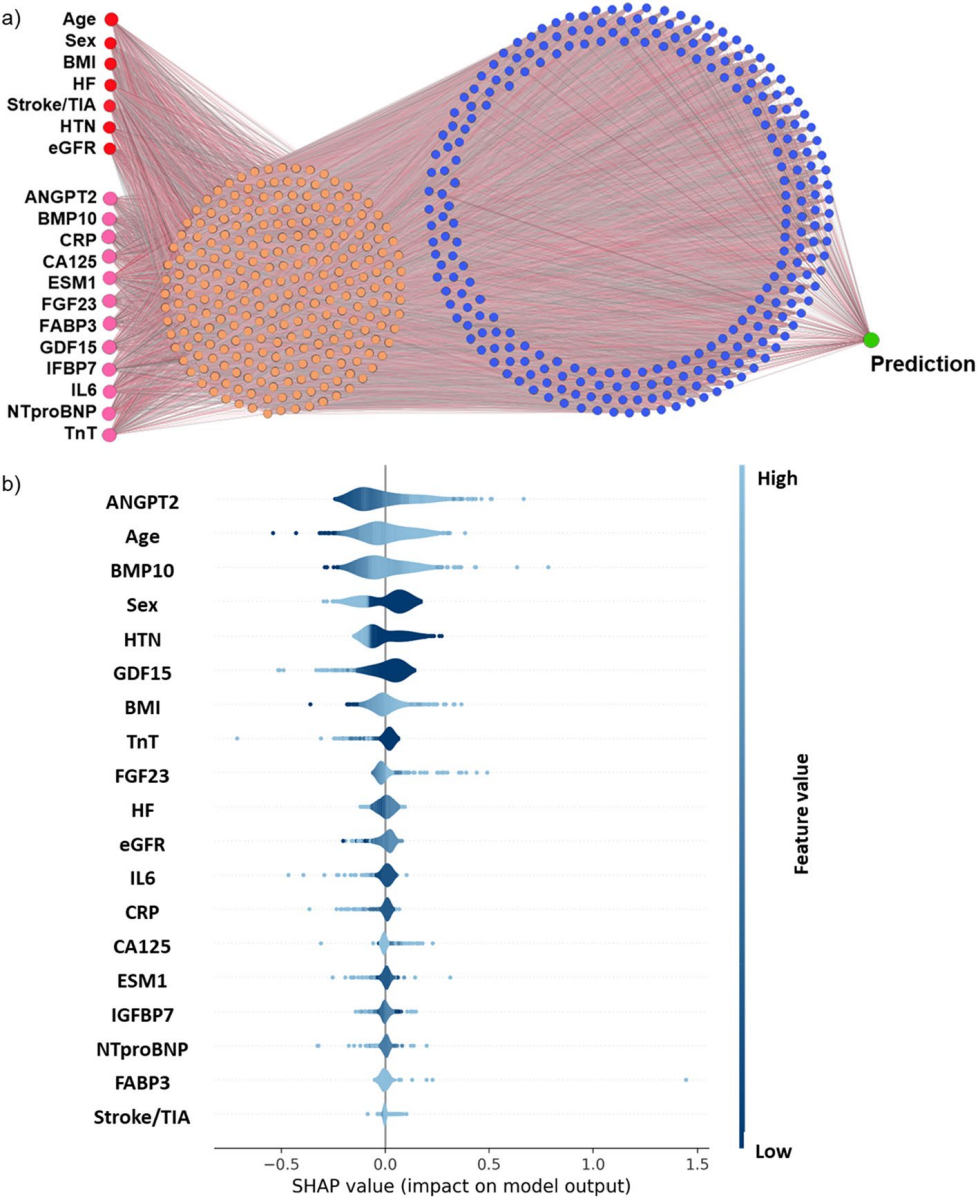
Neural network analysis confirm regression outcomes. (**a**) Illustration of the neural network architecture. Red and pink circles are the input variables. In orange and blue are the first and second hidden layer of the network, with increased abstraction. (**b**) Visualisation of the SHAP procedure using the validation cohort. Variables are ranked top to bottom from the most to least influential. Dark to light shading indicates low to high variable values. Negative and positive SHAP values correspond to tendencies towards sinus rhythm and AF respectively. *ANGPT2* angiopoietin 2, *BMI* body mass index, *BMP10* bone morphogenetic protein 10, *CRP* high-sensitivity C-reactive protein, *CA125* cancer antigen 125, *eGFR* estimated glomerular filtration rate, *ESM1* endothelial cell specific molecule 1, *FGF23* fibroblast growth factor 23, *FABP3* fatty acid binding protein 3, *GDF15* growth differentiation factor 15, *HF* heart failure, *HTN* hypertension, *IGFBP7* insulin like growth factor binding protein 7, *IL6* interleukin 6, *NTproBNP* N-terminal pro-B-type natriuretic peptide, *SHAP* Shapley additive explanations, *TIA* transient ischemic attack, *TnT* high-sensitivity cardiac troponin T.

The neural network was created using the discovery cohort with 2 layers of abstraction (visualised in Fig. 3a) and yielded an AUC (95% CI) of 0.784 (0.745, 0.822) in the validation cohort. In comparison with our previously published machine learning methodology, using random forest feature selection and fivefold cross-validation^12^ (Supplemental Material Fig. S2, Tables S6 and S7), neural networks yielded a better performance (AUC (95% CI) 0.784 (0.745, 0.822) compared to fivefold cross validation, AUC 0.733 (95% CI 0.691, 0.775)). The application of the SHAP procedure on the validation datasets calculated the influence of each variable on the model. The directionality of the top 3 clinical variables (age, sex, BMI) and the top 3 biomarkers (ANGPT2, BMP10, FGF23) associated with prevalent AF corresponded exactly with the regression modelling (Fig. 3b). The maximum variance inflation factor amongst the 19 variables entered into the model was 2.987, and therefore, multicollinearity was not relevant (Supplemental Material Table S8).

### Comparison between biomarkers

Using different methodologies to transform biomarkers may result in marginally different outcomes. As there is currently no accepted standard to transform biomarker data, we presented an alternative method and results as supplemental analyses to enable better comparison of our study outcomes with existing literature. We rank normalised each biomarker using the Blom transformation and described the relationship between biomarkers and the outcome per SD increase (Supplemental Material Figs. S3 and S4, Tables S9 and S10). Although this method may reduce the interpretability of an individual biomarker concentration, it provides a better comparison between biomarkers. Analyses using rank normalised biomarkers yielded very similar outcomes to the use of biomarkers as continuous variables. Age, sex, BMI, ANGPT2, and BMP10 were consistently selected despite different data transformations, however, the signals for NTproBNP and FGF23 were more inconsistent (Supplemental Material Fig. S4).

### Comparison with charge-AF score

The CHARGE-AF score is a clinical risk prediction model developed using data from a racially and geographically diverse population and therefore applicable for comparison in our cohort. The 5-year risk for the simple CHARGE-AF score^22^ was calculated as 1–0.9718412736^exp(ΣβX-12.5815600)^ in 1289 patients with complete data for all the variables (n = 196 with partially missing data). The C-statistic (95% CI) yielded was 0.631 (0.600, 0.661; sensitivity 78%, specificity 35%) as compared with 0.746 (0.719, 0.772; sensitivity 49%, specificity 83%) for the same patients using the present combined biomarker model (age, sex, BMI, BMP10, ANGPT2, FGF23) which also had better calibration (Supplemental Material Fig. S5).

### Comparison with NTproBNP

**Figure 4:**
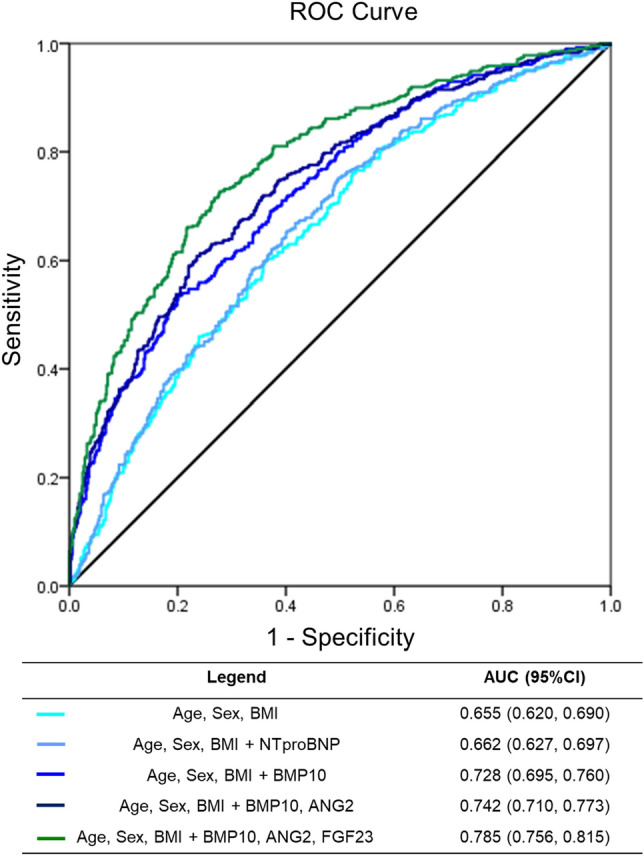
ANGPT2, BMP10, and FGF23 outperform NTproBNP. A comparison of model performance (using discovery cohort data) by area under the AUC and corresponding 95% CI demonstrating that (1) NTproBNP alone provides minimal improvements to the AUC in this cohort with known heart failure status, and (2) the incremental addition of biomarkers BMP10, ANGPT2, and FGF23 substantially improves the AUC. *AUC* area under the ROC curve, *CI* confidence intervals, *ANGPT2* angiopoietin 2, *BMP10* bone morphogenetic protein 10, *FGF23* fibroblast growth factor 23.

The addition of the established biomarker NTproBNP provided incremental improvement to classifications based only on age, sex, and BMI (AUC 0.655, 95% CI 0.620, 0.690 to AUC 0.665, 95% CI 0.627, 0.697). However, combining the biomarkers identified here (BMP10, ANGPT2, FGF23) yielded larger improvements than NTproBNP in this cohort with known heart failure status (Supplemental Material Table S4). The combination of all three biomarkers significantly improved the AUC (Fig. 4). We present the reclassification tables to demonstrate the effect of each additional biomarker (Supplemental Material Table S11). In a further step, we compared the combined biomarker model (age, sex, BMI, BMP10, ANGPT2, FGF23) with NTproBNP in patients ≥ 75 years old (n = 509; AF = 302, SR = 207) mirroring STROKESTOPII criteria and demonstrated that the biomarkers were better able to identify patients with AF compared to NTproBNP (Supplemental Material Fig. S6).

Type of patient enrolment (outpatient or inpatient), although showing some differences in clinical features with more heart failure and coronary artery disease in patients admitted) did not affect the analysis results (Data on file and Supplemental Material Table S12). We had not selected left atrial diameter as a marker for the primary model because it is not as readily available as age, sex and BMI, but requires a detailed echocardiography investigation, and we expected collinerality with NTproBNP. Indeed, in an analysis including left atrial size and left ventricular function, NTproBNP was eliminated (Data on file).

## Discussion

In unselected participants with cardiovascular conditions presenting to hospital, 3 simple clinical characteristics (age, sex, and BMI) and elevated concentrations of ANGPT2, BMP10, and FGF23 can identify participants with prevalent AF. Our findings illustrate the potential of quantifying the concentrations of several circulating biomolecules to enhance populations for patients with AF by combining a set of simple clinical characteristics and biomarkers. The results also highlight several disease relevant pathways that can be quantified in plasma and may be used to guide stratified prevention and therapy of AF. Our findings require external validation in prospective cohorts and potential updating or recalibration for a different setting.

The patients studied here resemble the high-risk populations that are currently considered for systematic and opportunistic AF screening^5,6^. Their clinical characteristics rendered them clear candidates for oral anticoagulation upon AF diagnosis. A particular characteristic is the acute care setting. This provides information on the interpretation of biomolecule concentrations in acutely ill patients, but also calls for validation in other settings, e.g. apparently healthy populations at risk for AF. The model exhibited a good performance and displayed high specificity (≥ 80%) in a cohort of patients enriched for cardiovascular conditions, rendering it useful in clinical settings such as hospitals or general practices. In our cohort, the model outperforms existing strategies to screen for AF using age alone^1,5,6^, age and cardiovascular comorbidities^1,5,6^, models integrating multiple clinical variables (CHARGE-AF)^22^, and age with one biomarker (BNP)^9^. The biomarker combination depicted offers the advantage of integrating different disease pathways and including at least one atrial-specific biomarker (BMP10), thus enabling differentiation for example between patients with AF and patients with heart failure^23^ or other conditions which may also elevate other cardiovascular biomarkers such as NTproBNP or TnT. Of note, both NT-proBNP^24^ and BMP10^25,26^ have recently been related to cardiovascular events in patients with AF.

Here, we took a pragmatic approach and assessed the ability of biomarkers to identify patients with prevalent AF, including 254 patients with diagnosed AF who were in sinus rhythm at the time of blood taking. Future studies should be planned to assess whether the novel biomolecules are elevated in prevalent and newly diagnosed AF, as well as the extent to which their elevation is related to the timing of arrhythmia episodes. Patients studied here presented with acute illness. This may affect the concentrations of biomarkers, calling for external validation in independent cohorts. This current approach is valuable for enriching screening, but does not evaluate risk of future incident AF, which was beyond the scope of our study. Future efforts to identify AF can use new technologies, providing more granularity for risk estimation which may be more advantageous compared to the conventional use of binary cutoff values.

**Figure 5:**
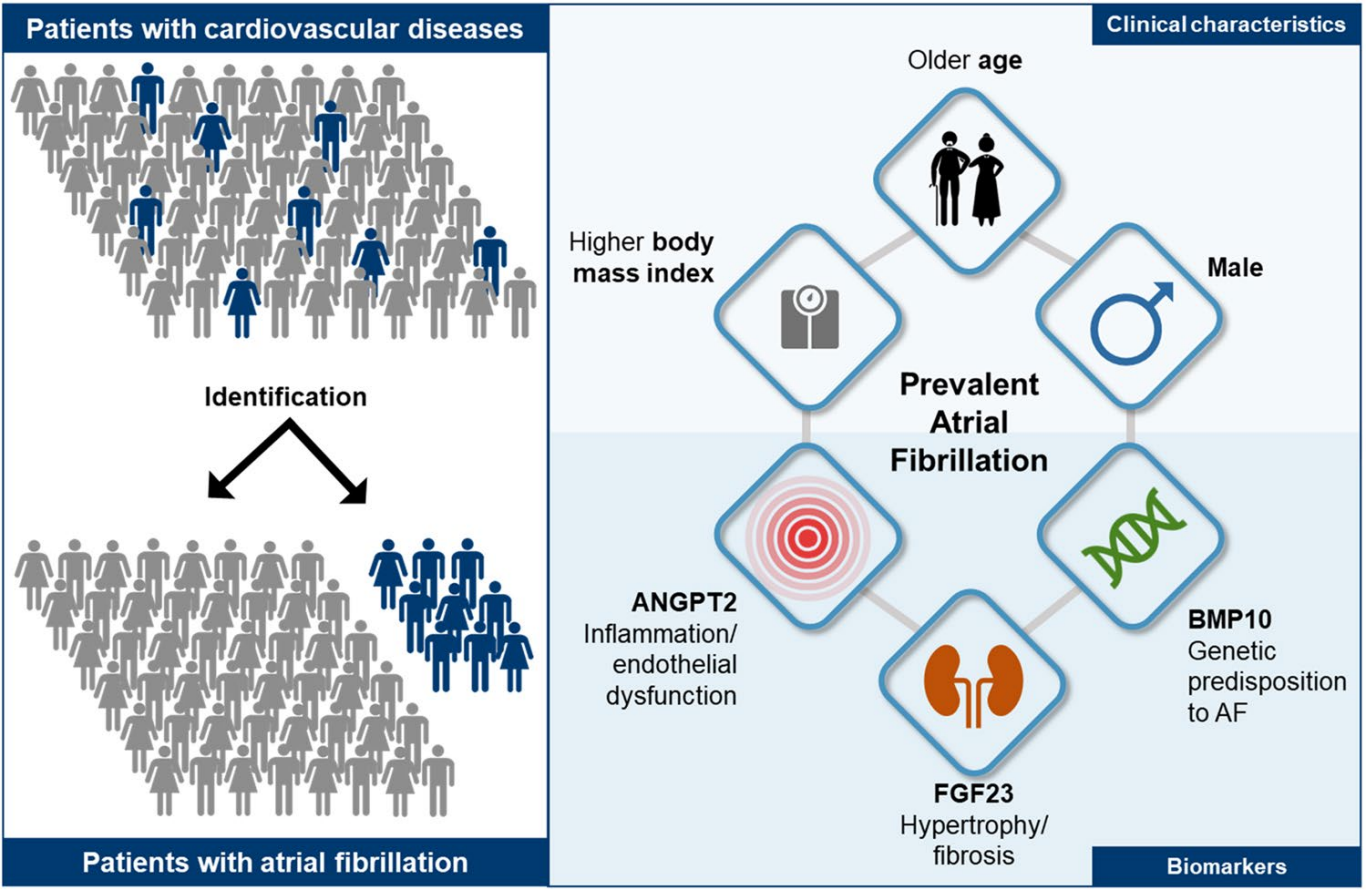
Detecting atrial fibrillation in patients with cardiovascular diseases. Combining age, sex, body mass index with ANGPT2, BMP10, and FGF23 discriminated between patients with and without prevalent AF. ANGPT2, BMP10, and FGF23 are circulating biomolecules representing distinct pathways associated with atrial cardiomyopathy and AF, namely hypertrophy and fibrosis (FGF23), endothelial dysfunction (ANGPT2), and the genomic predisposition to AF (BMP10). *ANGPT2* angiopoietin 2, *BMP10* bone morphogenetic protein 10, *FGF23* fibroblast growth factor 23.

Atrial fibrillation has different causes in different people. These causes of AF can interact and modify AF risk^15,27,28^. Reflecting the clinically suspected multifactorial aetiology of AF in unselected patients^27,28^, 9 of the 12 biomarkers tested showed elevated blood concentrations in patients with AF in our study (ANGPT2, BMP10, CA125, ESM1, FABP3, FGF23, GDF15, IGFBP7, and NTproBNP), confirming prior reports. Using several analytical methods and evaluations of 12 different biomarkers, increased concentrations of three biomarkers were most strongly associated with AF (BMP10, ANGPT2, and FGF23; Fig. 5). Interestingly, these three biomolecules are expressed in three different cell types (BMP10: cardiomyocytes; ANGPT2: endothelial cells; FGF23: osteocytes) and might represent three underlying molecular mechanisms of AF.

Mutations in BMP10, a polypeptide encoded by the *BMP10* gene belonging to the TGF-β superfamily have been associated with cardiovascular disease^29^. In humans, BMP10 expression is enriched in the right atrium and is increased in diseased atria^30^. As BMP10 is uniquely expressed in cardiomyocytes^31^ and restricted to atria^32^, it is a promising atrial-specific biomarker in circulating blood. Elevated BMP10 in blood have been shown to be predictive of recurrent AF after ablation for AF in patients^30^. Downregulation of the paired-like homeodomain transcription factor 2 (PITX2) or an enhancer region close to the common AF gene variants is associated with increased left atrial expression of the *BMP10* gene^30^. There have been recent work reporting the use of BMP10 for predicting AF recurrences^33,34^ and ischemic stroke risk^25^, however, further work on the atrial effects of BMP10 is warranted.

ANGPT2 is a bioactive growth factor belonging to the angiopoietin/Tie (tyrosine kinase with Ig and EGF homology domain) family of signalling proteins that play a major role in maintaining vascular homeostasis. Located and synthesised by endothelial cells, it is rapidly released in inflammation or vascular damage^35^. In its active form, ANGPT2 can act on its receptor Tie2 in an autocrine manner to promote endothelial barrier disassembly and leukocyte extravasation^36^. This may acutely alter atrial function and lead to structural atrial remodelling in the long-term^15^. Elevated blood concentrations of ANGPT2 were found in patients with chronic AF^37^. A greater understanding of the role of ANGPT2 for AF at the cellular level is required to understand how ANGPT2/Tie2 signalling in cardiac endothelium regulates cardiac remodelling.

FGF23 is a hormone secreted by osteocytes in the bone and functions to regulate phosphate homeostasis. Elevation of circulating FGF23 in patients with chronic kidney disease has been associated with increased risk of cardiovascular disease, including AF, by promoting cardiac remodelling^13^. There is biological plausibility for a causal relationship between FGF23 and left ventricular hypertrophy and atrial fibrosis, which is often observed in patients with AF^38^. FGF23 has been consistently associated with prevalent AF^12,13^ and our data supports FGF23 as a biomarker for AF and AF-related mechanisms.

### Limitations

The inclusion criteria for BBC-AF were broad, aiming to represent unselected patients presenting to hospital for care. While this may be viewed as a strength for researchers and clinicians seeking to identify patients presenting with AF in acute settings, the process also induces bias in patient selection and will affect biomarker concentrations compared to e.g. screening of apparently healthy individuals. Therefore, we have extensively described the characteristics of the cohort that should be read in parallel with the study outcomes. The single-centre setting enabled unified phenotyping but necessitates validation in external cohorts. Therefore, external validation in prospective cohorts with long-term follow-up for incident AF and in population-based cohorts is desirable. We also advise caution in like-for-like comparison of outcomes with other published scores as differences will exist between study designs (case–control vs population-based). Additional analyses based on our data and on external validation will also be needed to determine the cost-effectiveness of quantifying three biomarkers (e.g. compared with collecting multiple clinical variables for the CHARGE-AF score or using 7-day ambulatory monitoring). The directionality of influence of the biomarkers need to be interpreted in accordance with the characteristics of the cohort; e.g. the influence of markers such as TnT, hsCRP, and GDF15 reflect the patterns of co-morbidities present in the sinus rhythm group which have a higher proportion of patients with diabetes, coronary artery disease, and hypertension. While systematic 7-day Holter monitoring for undiagnosed AF is a strength of this cohort compared to other observational data sets, longer monitoring periods are likely to identify even more patients with rare atrial arrhythmias^39^. The therapeutic consequences of very rare arrhythmias were evaluated, e.g. in the controlled NOAH-AFNET 6 and ARTESiA trials^39–41^, with NOAH-AFNET 6 results showing a low stroke risk in patients with atrial high-rate episodes (AHRE).

## Conclusion

Our study recapitulates age, sex, and BMI, as clinical markers for AF. Elevated ANGPT2, BMP10 and FGF23, are novel biomarkers that were robustly associated with AF in this study. Results suggest that age and different disease processes approximated by these three biomolecules contribute to AF in patients. In an acute care setting, a stratification procedure using age, sex, BMI, and these three biomarkers can identify people at high risk of prevalent AF and calls for external validation.

### Supplementary Information

Supplementary Information.

## Data Availability

The data underlying this article cannot be shared publicly due to the privacy of individuals who participated in the study. Reasonable requests to the corresponding author for data access will be considered.
