# Supplementary material for: An angiopoietin 2, FGF23, and BMP10 biomarker signature differentiates atrial fibrillation from other concomitant cardiovascular conditions
Source: Sci Rep. 2023 Oct 5;13:16743. doi: 10.1038/s41598-023-42331-7 (PMC10556075; doi:10.1038/s41598-023-42331-7)
Supplement: Supplementary file 1 — Supplementary Information. [file 41598_2023_42331_MOESM1_ESM.docx]

**SUPPLEMENTAL MATERIAL**

**An angiopoietin 2, FGF23, and BMP10 biomarker signature differentiates atrial fibrillation from other concomitant cardiovascular conditions.**

**AUTHORS**

Winnie Chua^1^ PhD, Victor R. Cardoso^1,2,3^ PhD, Eduard Guasch^4^ MD, PhD, Moritz F. Sinner^5,6^ MD, MPH, Christoph Al-Taie^7, 8, 9^, Paul Brady^1,10^ MD, Barbara Casadei^11^ MD, DPhil, Harry JGM Crijns^12^ MD, PhD, Elton AMP Dudink^12^ MD, PhD, Stéphane N. Hatem^13^ MD, PhD, Stefan Kääb^5,6^ MD, PhD, Peter Kastner^14^ PhD, Lluis Mont^4^ MD, PhD, Frantisek Nehaj^1,10^ MUDr, Yanish Purmah^1,10^ MD, Jasmeet S. Reyat^1^ PhD, Ulrich Schotten^12^ MD, PhD, Laura C Sommerfeld^1,7, 8, 9^ PhD, Stef Zeemering^12^ PhD, André Ziegler^15^ PhD, Georgios V. Gkoutos^2,3^ PhD, Paulus Kirchhof^1,8,9^ MD, Larissa Fabritz^1,7,8,9^ MD.

^1^ Institute of Cardiovascular Sciences, University of Birmingham, Birmingham, UK.
^2^ MRC Health Data Research UK (HDR), Midlands Site, UK.

^3^ Institute of Cancer and Genomic Sciences, University of Birmingham, Birmingham, UK.

^4^ Hospital Clinic de Barcelona; Institute of Biomedical Research August Pi Sunyer (IDIBAPS), Barcelona, ES.

^5^ Department of Medicine I, University Hospital, LMU, Munich, DE.

^6^ German Centre for Cardiovascular Research (DZHK), partner site: Munich Heart Alliance, Munich, DE.

^7^ University Center of Cardiovascular Science, University Heart and Vascular Center Hamburg, University Medical Center Hamburg-Eppendorf, DE.

^8^ German Centre for Cardiovascular Research (DZHK), partner site: Hamburg/Kiel/Lübeck, DE.

^9^ Department of Cardiology, University Heart and Vascular Center Hamburg, University Medical Center Hamburg-Eppendorf, DE.

^10^ Sandwell and West Birmingham Hospitals NHS Trust, Birmingham, UK.

^11^ University of Oxford, Oxford, UK.
^12^ Cardiovascular Research Institute Maastricht (CARIM), Maastricht University, Maastricht, NL.

^13^ IHU-ICAN Institute of Cardiometabolism and Nutrition, Paris, FR.

^14^ Roche Diagnostics GmbH, Penzberg, DE.
^15^ Roche Diagnostics International AG, Rotkreuz, CH.

**SUPPLEMENTAL METHODS AND RESULTS**

Machine learning algorithms references:

1. Keras
2. Scikit-learn
3. RELU activation
4. dropout layer
5. adam optimizer
6. Shapley Additive exPlanations (SHAP)
7. Chollet, François, al. e. Keras. 2015.
8. Pedregosa F, Varoquaux G, Gramfort A, et al. Scikit-learn: Machine Learning in Python. J Mach Learn Res 2011; 12: 2825-30.
9. Nair V, Hinton GE. Rectified linear units improve restricted boltzmann machines. Proceedings of the 27th International Conference on International Conference on Machine Learning. Haifa, Israel: Omnipress; 2010. p. 807-14.
10. Srivastava N, Hinton G, Krizhevsky A, Sutskever I, Salakhutdinov R. Dropout: A Simple Way to Prevent Neural Networks from Overfitting. J Mach Learn Res 2014; 15: 1929-58.
11. Kingma DP, Ba J. Adam: A method for stochastic optomization. Conference paper at the 3rd International Conference for Learning Representations, San Diego 2017.
12. Lundberg SM, Lee SI. A Unified Approach to Interpreting Model Predictions. Adv Neur In 2017; 30.

Biomarker assays:

Six proteins, CA125, Growth Differentiation Factor-15 (GDF15), Interleukin-6 (IL6), N‑terminal pro B‑type natriuretic peptide (NTproBNP), cardiac Troponin T (TnT), and Cardiac C-Reactive Protein (CRP), were measured using commercially available Roche immunoassays (cobas Elecsys® CA 125 II, GDF-15, IL-6, NTproBNP II, high sensitivity Troponin T; cobas c 501 for high sensitivity CRP). A further 6 proteins, angiopoietin 2 (ANGPT2), bone morphogenetic protein 10 (BMP10), endothelial specific molecule 1 (ESM1), fatty acid binding protein 3 (FABP3), fibroblast growth factor 23 (FGF23), and insulin-like growth factor binding protein 7 (IGFBP7) were quantified using pre-commercial Elecsys® immunoassays. Biomarkers were quantified at Roche Diagnostics, Mannheim, Germany.

The sandwich-immunoassays used were developed by applying monoclonal antibodies specifically screened for the detection of the respective target.

**1. MACHINE LEARNING (5-FOLD CROSS-VALIDATION).**

**Methods:** Data were separated into training and test sets using the same patients as the regression model. A 5-fold validation approach with ROSE sampling algorithm was used for model creation. The process was repeated 5 times using different patients in each fold, leading to different models for improved robustness. The algorithms employed were: Lasso and elastic-net regularized generalized linear model, Support vector machines with linear Kernel, Random forest, Stochastic gradient boosting and Recursive partitioning. In a secondary comparison, the number of appearances in the top 5 important variables was counted.

**Results:** The analysis concluded with a total of 25 different models. The best performing model on our 5-fold validation stage was Support vector machines with linear Kernel with a resulting AUC of 0.733 (95% CI 0.691, 0.775; **table S5, fig S1**). Note the elevated importance of BMP10 and ANGPT2 to most created models (**table S6**). Directionality of influence cannot be determined from this ranking.

**2. ANALYSIS OF TRANSFORMED BIOMARKERS.**

**Methods:** Biomarkers are best analysed in a continuous form, using units in which they were originally quantified. Retaining the data in this way as opposed to transforming the data gives the advantage of fidelity and granularity for reflecting the effect of biomarkers on the outcome as closely as possible. However, as biomarker assays have varying measurement ranges and sensitivities (e.g. measurement range for NTproBNP 5 to 35000 pg/mL, for BMP10 1 to 5 ng/mL), it is important to acknowledge that these inherent properties of the assays may make comparing between biomarkers a challenge. As an alternative analysis, we have therefore considered the biomarkers by using rank normalised Blom transformation. We present the analysis using this transformation and describe the effect of biomarkers per SD increase.

**Results: Regression Analysis.** In summary, the outcome for univariate (**fig s2**) and multivariate analyses using Blom transformed biomarkers mirrored the outcomes of using biomarkers as continuous variables with minor differences in odds ratios and AUCs. In the backward elimination, 1 additional variable remained in the model (NTproBNP, **table S8**). Validation of the model using the bootstrapped coefficients (**table S9**) in the validation cohort yielded an identical AUC to the main analysis (0.724, 95%CI 0.681, 0.766).

**Machine Learning.** Using Blom transformed biomarkers, the AUC (95%CI) for the model was higher 0.809 (0.809, 0.810) with similar variable rankings to the biomarker data (**fig S3**).

**3. COMPARISON WITH CHARGE-AF RISK SCORE.**

**Methods:** The performance of the biomarker was compared with the simple CHARGE-AF risk score. The CHARGE-AF risk score was calculated for all patients who have complete data for the 11 variables in the score (n = 1289). The score was calculated as follows: [0.508 × age (5 years)] + [0.465 x race (white)] + [0.248 × height (10 cm)] + [0.115 × weight (15kg)] + [0.197 × systolic blood pressure (20 mm Hg)] – [0.101 × diastolic blood pressure (10 mm Hg)] + [0.359 × current smoker] + [0.349 × antihypertensive medication] + [0.237 × diabetes] + [0.701 × congestive heart failure] + [0.496 × myocardial infarction]. The coefficients for each risk factor are from the derivation study for the CHARGE-AF risk score. The AUC and calibration plots comparing the CHARGE-AF risk score and the biomarker model were calculated.

**Results:** Presented in **fig S4.**

**SUPPLEMENTAL TABLES**

| **Selected variable** | **Β coefficient** | **Odds ratio**  (OR) | **95% confidence interval** | | **P-value** |
| --- | --- | --- | --- | --- | --- |
|  |  |  | Lower | Upper |  |
| **Age** (year) | 0.050 | 1.051 | 1.036 | 1.066 | <0.001 |
| **Sex** (female) | -0.672 | 0.511 | 0.371 | 0.703 | <0.001 |
| **BMI** (kg/m^2^) | 0.046 | 1.047 | 1.021 | 1.073 | <0.001 |
| **Hypertension** | -0.520 | 0.594 | 0.438 | 0.806 | 0.001 |
| **ANGPT2** (ng/mL) | 0.148 | 1.160 | 1.083 | 1.242 | <0.001 |
| **BMP10** (ng/mL) | 1.050 | 2.858 | 2.039 | 4.005 | <0.001 |
| **ESM1** (ng/mL) | -0.070 | 0.932 | 0.858 | 1.013 | 0.099 |
| **TnT** (100 pg/mL) | -0.032 | 0.968 | 0.947 | 0.990 | 0.005 |
| **GDF15** (100 pg/mL) | -0.022 | 0.979 | 0.971 | 0.986 | <0.001 |
| **FGF23** (100 pg/mL) | 0.031 | 1.032 | 1.007 | 1.058 | 0.013 |
| Constant | -6.621 | 0.001 | - | - |  |

**Table S1. Variables identified by backwards elimination to be associated with higher odds of AF.** Odds ratio (OR) and 95% confidence intervals (CI) were quantified using logistic regression. For sex, male was the reference category. AF, atrial fibrillation; BMI, body mass index; ANGPT2, angiopoietin 2; BMP10, bone morphogenetic protein 10; CRP, C-reactive protein; FGF23, fibroblast growth factor 23; GDF15, growth differentiation factor 15; NTproBNP, N-terminal pro-B-type natriuretic peptide; TnT, high-sensitivity cardiac troponin T.

| **Selected variable** | **Fitted model** | | | | **Bootstrapped model** | | | |
| --- | --- | --- | --- | --- | --- | --- | --- | --- |
|  | **Β coefficient** | **Odds ratio**  (OR) | **95% confidence interval** | | **Β coefficient** | **Odds ratio**  (OR) | **95% confidence interval** | |
|  |  |  | Lower | Upper |  |  | Lower | Upper |
| **Age** (years) | 0.036 | 1.037 | 1.023 | 1.051 | 0.036 | 1.037 | 1.023 | 1.054 |
| **Sex** (female) | -0.440 | 0.644 | 0.476 | 0.870 | -0.430 | 0.651 | 0.490 | 0.824 |
| **BMI** (kg/m^2^) | 0.036 | 1.037 | 1.013 | 1.061 | 0.036 | 1.037 | 1.013 | 1.061 |
| **ANGPT2** (ng/mL) | 0.113 | 1.120 | 1.053 | 1.191 | 0.111 | 1.117 | 1.054 | 1.206 |
| **BMP10** (ng/mL) | 0.717 | 2.048 | 1.530 | 2.743 | 0.715 | 2.044 | 1.548 | 2.846 |
| **FGF23** (100 pg/mL) | 0.008 | 1.008 | 0.986 | 1.031 | 0.004 | 1.004 | 0.989 | 1.057 |
| Constant | -5.723 | 0.003 | - | - | -5.723 | 0.003 | - | - |

**Table S2. Optimism-adjusted model:** **Three clinical characteristics (age, sex, BMI) and three biomarkers (ANGPT2, BMP10, FGF23) predicted prevalent AF**. Variables were also bootstrapped to account for potential overfitting. For sex, male was the reference category.

AF, atrial fibrillation; BMI, body mass index; ANGPT2, angiopoietin 2; BMP10, bone morphogenetic protein 10; FGF23, fibroblast growth factor 23.


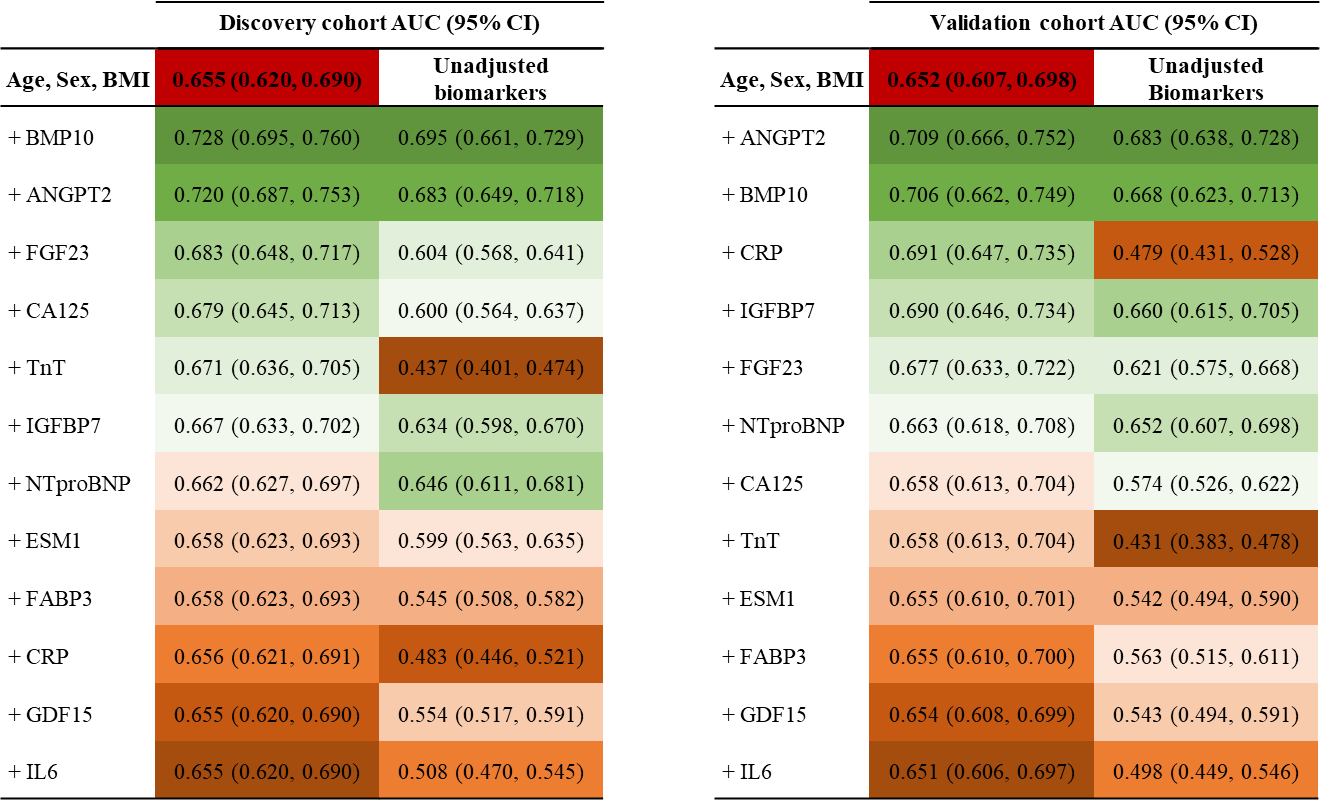


**Table S3: Incremental effect of individual biomarkers to predict AF.** Model performance (by area under the ROC curve, AUC and 95% confidence intervals, CI) for individual biomarkers in addition to clinical characteristics (Age, Sex, body mass index, BMI) in the discovery (left) and validation (right) cohorts. Biomarkers are ranked from the highest (green) to lowest (brown) improvement. Unadjusted biomarkers describe the AUC of the individual biomarker only.

ANGPT2, angiopoietin 2; BMP10, bone morphogenetic protein 10; CRP, high-sensitivity C-reactive protein; CA125, cancer antigen 125; ESM1, endothelial cell specific molecule 1; FGF23, fibroblast growth factor 23; FABP3, fatty acid binding protein 3; GDF15, growth differentiation factor 15; IGFBP7, insulin like growth factor binding protein 7; IL6, interleukin 6; NTproBNP, N-terminal pro-B-type natriuretic peptide; TnT, high-sensitivity cardiac troponin T.


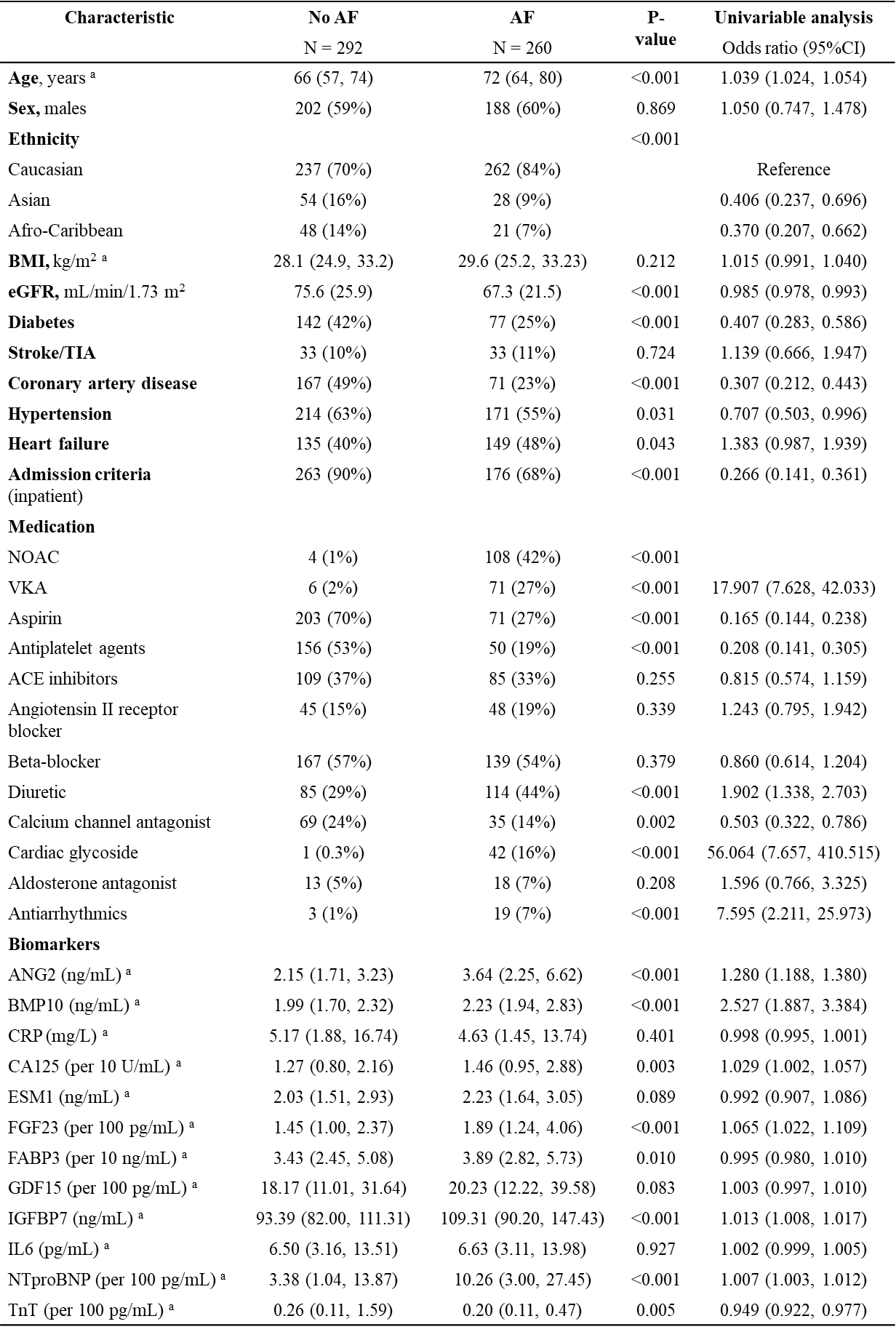


**Table S4: Patient characteristics of the validation cohort.** Categorical variables are reported as n (%), continuous variables are reported as mean (standard deviation) or median (interquartile range) for skewed distributions (^a^). The independent t-test (or Mann-Whitney U test for non-parametric distributions) and Χ^2^ tests were used to compare characteristics between patients.

BMI, body mass index; eGFR, estimated glomerular filtration rate; TIA, transient ischemic attack; NOAC, non-vitamin K antagonist oral anticoagulant; VKA, vitamin K antagonist; ACE, angiotensin-converting enzyme; ANGPT2, angiopoietin 2; BMP10, bone morphogenetic protein 10; CRP, high-sensitivity C-reactive protein; CA125, cancer antigen 125; ESM1, endothelial cell specific molecule 1; FGF23, fibroblast growth factor 23; FABP3, fatty acid binding protein 3; GDF15, growth differentiation factor 15; IGFBP7, insulin like growth factor binding protein 7; IL6, interleukin 6; NTproBNP, N-terminal pro-B-type natriuretic peptide; TnT, high-sensitivity cardiac troponin T.

| **Variable** | **Cut-offs** (%, 95%CI) | | | |
| --- | --- | --- | --- | --- |
|  | **10%** | **20%** | **30%** | **40%** |
| **Sensitivity** (%) | 99.77 (98.73, 99.99) | 96.56 (94.39, 98.06) | 85.55 (81.89, 88.71) | 70.87 (66.36, 75.10) |
| **Specificity** (%) | 1.61 (0.74, 3.04) | 14.70 (11.86, 17.91) | 41.22 (37.10, 45.43) | 62.01 (57.84, 66.05) |
| **Positive predictive value** (%) | 44.21 (40.75, 47.01) | 46.93 (45.97, 47.90) | 43.21 (51.23, 55.18) | 59.31 (56.34, 62.21) |
| **Negative predictive value** (%) | 90.00 (53.37, 98.61) | 84.54 (76.18, 90.33) | 78.50 (74.00, 82.40) | 73.15 (69.89, 76.18) |
| **Overall accuracy** (%) | 44.67 (41.55, 47.82) | 50.60 (47.45, 53.76) | 60.66 (57.55, 63.72) | 65.85 (62.85, 68.84) |
| **Predictions by the biomarker model** | | | | |
| **Patients “at risk”** (n) | 984 | 897 | 701 | 521 |
| **Identified** (“at risk”, with AF; n) | 435 | 421 | 373 | 309 |
| **Patients “not at risk”** (n) | 10 | 97 | 293 | 473 |
| **Identified** (“not at risk”, no AF; n) | 9 | 82 | 230 | 346 |
| **Missed** (“not at risk”, with AF, n) | 1 | 15 | 63 | 127 |

**Table S5. Model metrics for different thresholds.** Performance of the optimism-adjusted model at different cut-off thresholds.


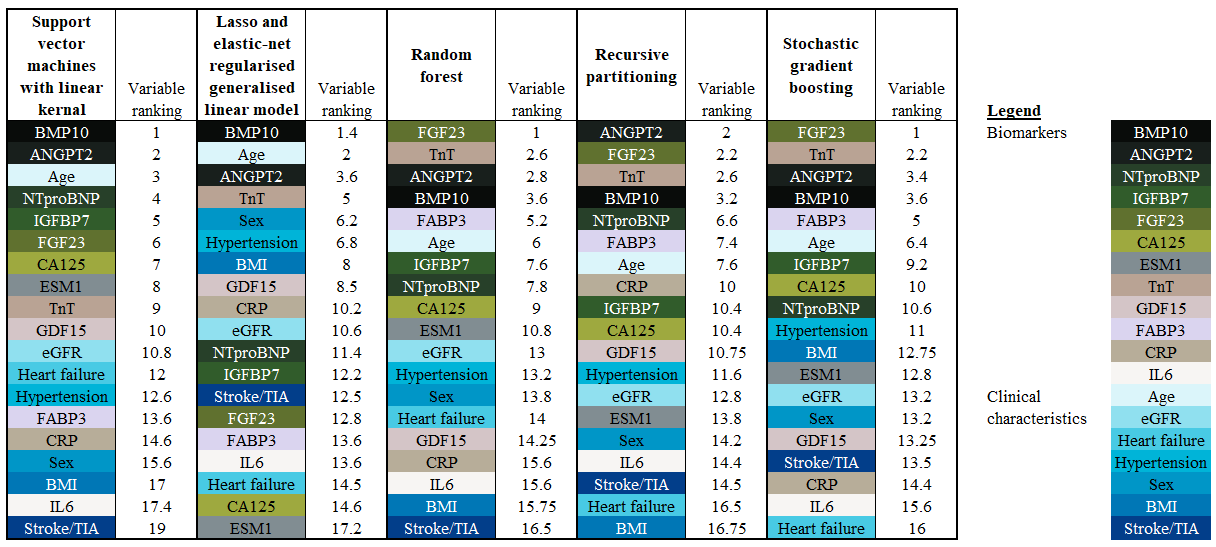


**Table S6: Detecting AF using machine learning.** Five machine learning algorithms were used to train the data. The ordered importance of the different variables for the different algorithms. BMI, body mass index; eGFR, estimated glomerular filtration rate; TIA, transient ischemic attack; HF, heart failure; HTN, hypertension; ANGPT2, angiopoietin 2; BMP10, bone morphogenetic protein 10; CRP, high-sensitivity C-reactive protein; CA125, cancer antigen 125; ESM1, endothelial cell specific molecule 1; FGF23, fibroblast growth factor 23; FABP3, fatty acid binding protein 3; GDF15, growth differentiation factor 15; IGFBP7, insulin like growth factor binding protein 7; IL6, interleukin 6; NTproBNP, N-terminal pro-B-type natriuretic peptide; TnT, high-sensitivity cardiac troponin T.


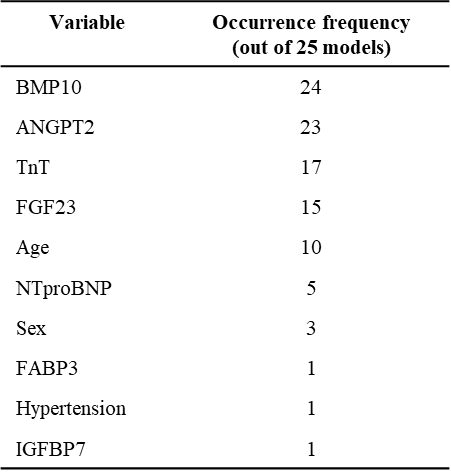


**Table S7: Most common clinical and biomarker variables for AF detection identified by the algorithms.** Aggregated frequency of variables appearing in 25 model iterations. Note the elevated importance of BMP10 and ANGPT2 to most created models. Directionality of influence cannot be determined from this ranking. It should be understood in the context of the baseline characteristics in this cohort. ANGPT2, angiopoietin 2; BMP10, bone morphogenetic protein 10; CRP, C-reactive protein; FABP3, fatty acid binding protein 3; FGF23, fibroblast growth factor 23; GDF15, growth differentiation factor 15; IGFBP7, insulin growth factor binding protein 7; NTproBNP, N-terminal pro-B-type natriuretic peptide; TnT, high-sensitivity cardiac troponin T.

**
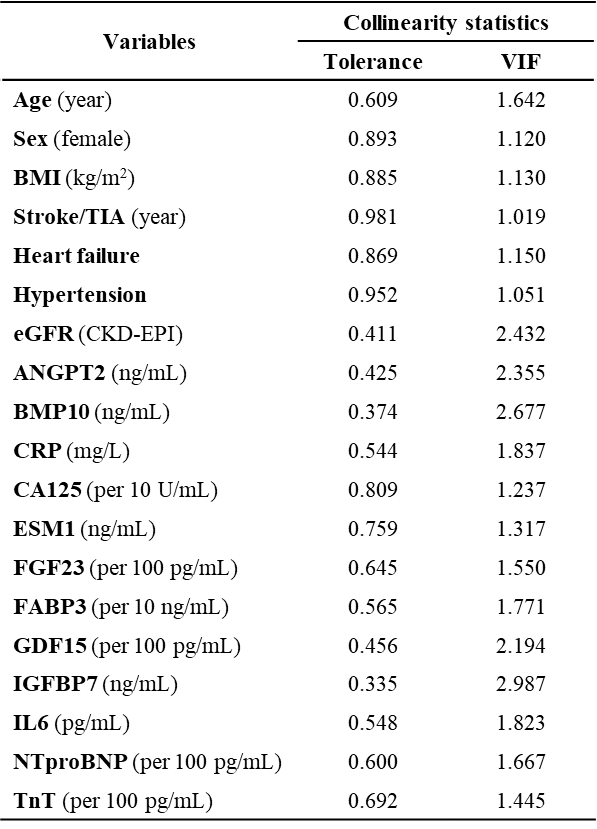
**

**Table S8**: **Collinearity statistics variables considered in the modelling.** The tolerance and variance inflation factor (VIF) for variables included, BMI, body mass index; eGFR, estimated glomerular filtration rate; TIA, transient ischemic attack; NOAC, non-vitamin K antagonist oral anticoagulant; VKA, vitamin K antagonist; ACE, angiotensin-converting enzyme; ANGPT2, angiopoietin 2; BMP10, bone morphogenetic protein 10; CRP, high-sensitivity C-reactive protein; CA125, cancer antigen 125; ESM1, endothelial cell specific molecule 1; FGF23, fibroblast growth factor 23; FABP3, fatty acid binding protein 3; GDF15, growth differentiation factor 15; IGFBP7, insulin like growth factor binding protein 7; IL6, interleukin 6; NTproBNP, N-terminal pro-B-type natriuretic peptide; TnT, high-sensitivity cardiac troponin T.


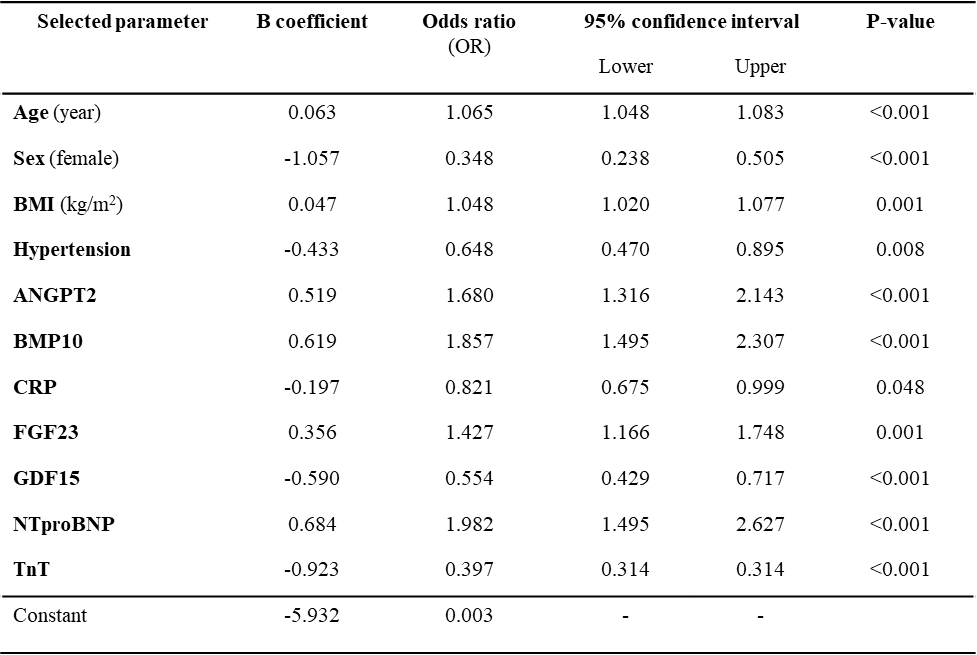


**Table S9:** **Initial selection of variables with higher odds of AF.** Apparent model of parameters identified by backward selection as associated with higher odds of AF. Odds ratios for Blom transformed biomarkers are per SD increase. For sex, female was the reference category. AF, atrial fibrillation; BMI, body mass index; ANGPT2, angiopoietin 2; BMP10, bone morphogenetic protein 10; CRP, C-reactive protein; FGF23, fibroblast growth factor 23; GDF15, growth differentiation factor 15; NTproBNP, N-terminal pro-B-type natriuretic peptide; TnT, high-sensitivity cardiac troponin T.


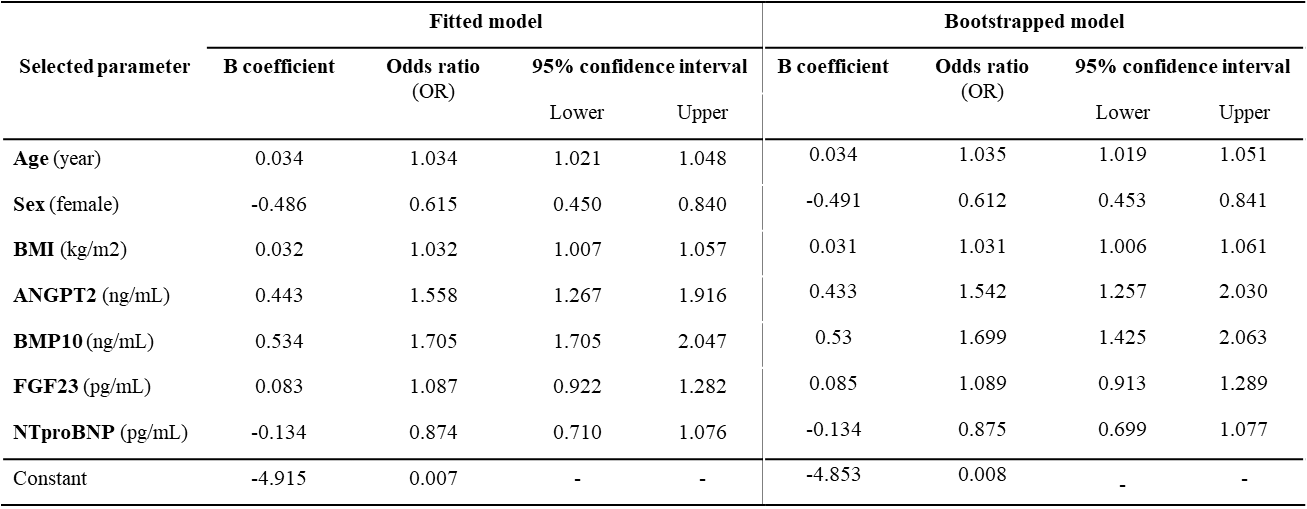


**Table S10: Optimism-adjusted model for prevalent AF.** Three clinical characteristics (age, sex, BMI) and three biomarkers (ANGPT2, BMP10, FGF23) predicted prevalent AF. Variables were also bootstrapped to account for potential overfitting. Coefficients for the fitted as well as optimism-adjusted model. For sex, female was the reference category. Odds ratios for Blom transformed biomarkers are per SD increase. AF, atrial fibrillation; BMI, body mass index; ANGPT2, angiopoietin 2; BMP10, bone morphogenetic protein 10; FGF23, fibroblast growth factor 23.


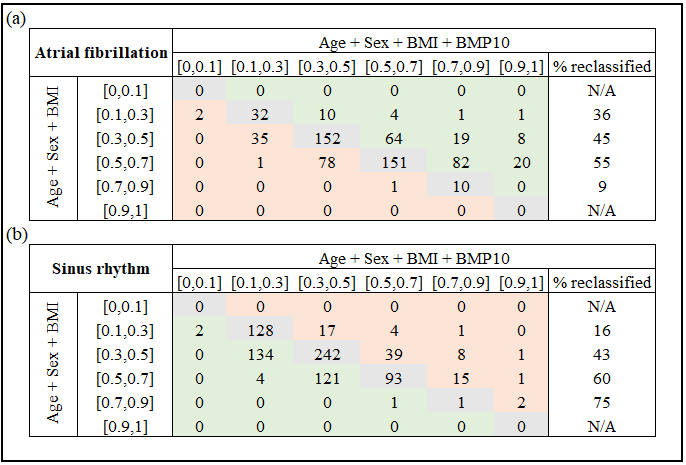

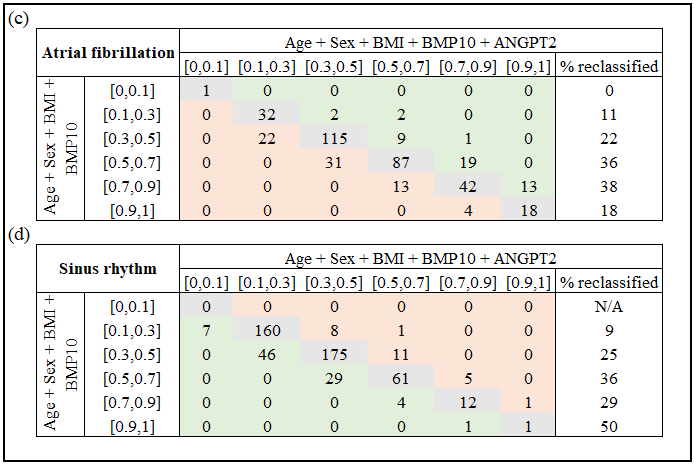


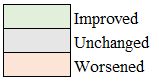


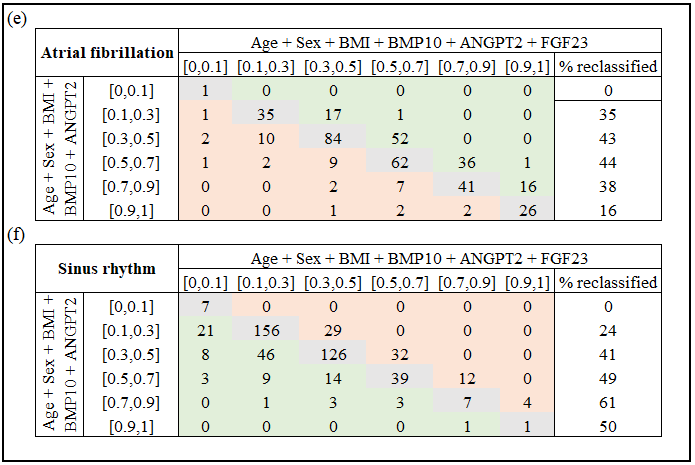


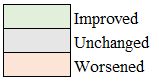


**Table S11: Improvement of reclassification with additional biomarkers.** The incremental change in classification for each additional biomarker on top of age, sex, and body mass index (BMI) are compared for BMP10 (a and b), BMP10 + ANGPT2 (c and d) and BMP10 + ANGPT2 + FGF23 (e and f). For each table, predictive probabilities are stratified into bins. Improvements in predictive probabilities towards a correct classification are shaded in green, where an improvement for patients with atrial fibrillation is defined as an increase in predictive probability, and an improvement for patients in sinus rhythm is defined as a decrease in predictive probability. Conversely, predictive probabilities which worsen are shaded in orange, defined as a decrease in predictive probability for patients with atrial fibrillation, and an increase in predictive probability for patients in sinus rhythm. Predictive probabilities which remain within the same bin are shaded in grey.

|  | **Inpatient** | **Outpatient** | **P-Value** |
| --- | --- | --- | --- |
| **n** | 468 | 115 |  |
| **Age**, years ^a^ | 68.9 (12) | 68.4 (13) | 0.728 |
| **Sex**, male | 286 (61%) | 66 (57%) | 0.532 |
| **Ethnicity** |  |  | 0.261 |
| Afro-Caribbean | 53 (11%) | 8 (7%) |  |
| Asian | 63 (13%) | 20 (17%) |  |
| Caucasian | 352 (75%) | 87 (75%) |  |
| **BMI**, kg/m2 ^a^ | 29.5 (6.4) | 30.4 (5.9) | 0.201 |
| **eGFR** mL/min/1.73m^2^ | 69.9 (23.6) | 73.5 (20.5) | 0.101 |
| **Diabetes** | 185 (40%) | 27 (24%) | 0.002 |
| **Stroke/TIA** | 42 (9%) | 11 (10%) | 0.987 |
| **Coronary artery disease** | 197 (42%) | 19 (16%) | <0.001 |
| **Hypertension** | 274 (60%) | 74 (64%) | 0.303 |
| **Heart failure** | 253 (54%) | 30 (26%) | <0.001 |
| **diastolic LA diameter** | 41.3 (9.0) | 40.0 (11.5) | 0.265 |
| **LVEF** | 53.5 (15.8) | 59.8 (10.5) | <0.001 |
| **AF** | 185 (40%) | 87 (76%) | <0.001 |
| **Biomarkers** |  |  |  |
| ANGPT2 (ng/mL) ^a^ | 4.0 (1.88, 5.2) | 3.0 (1.72, 3.63) | <0.001 |
| BMP10 (ng/mL) ^a^ | 2.3 (1.79, 2.69) | 2.2 (1.89, 2.53) | 0.086 |
| CA125 (U/mL) ^a^ | 35.7 (8.65, 25.54) | 13.9 (7.68, 16.51) | <0.001 |
| CRP (mg/L) ^a^ | 31.4 (2.39, 20.56) | 3.7 (0.71, 4.35) | <0.001 |
| ESM1 (ng/mL) ^a^ | 2.9 (1.64, 3.34) | 2.0 (1.45, 2.33) | <0.001 |
| FABP3 (ng/mL) ^a^ | 65.9 (27.49, 61.71) | 35.9 (27.06, 39.41) | 0.001 |
| FGF23 (pg/mL) ^a^ | 369.6 (115.77, 329.05) | 245.4 (120.54, 219.98) | 0.003 |
| GDF15 (pg/mL) ^a^ | 3093.3 (1271.75, 3728.75) | 1976.7 (1085.5, 2572) | <0.001 |
| IGFBP7 (ng/mL) ^a^ | 113.0 (84.53, 123.78) | 112.2 (89.98, 121.65) | 0.848 |
| IL6 (pg/mL) ^a^ | 15.1 (4.28, 16.67) | 4.8 (2.05, 4.82) | <0.001 |
| NTproBNP (pg/mL) ^a^ | 2482.8 (231.15, 2817.25) | 641.8 (82.66, 926.1) | <0.001 |
| TnT (pg/mL) ^a^ | 456.1 (14.35, 106.05) | 14.8 (7.64, 17.5) | <0.001 |

**Table S12: Patient characteristics of inpatients and outpatients with full echocardiography assessment.** Categorical variables are reported as n (%), continuous variables are reported as mean (standard deviation) or median (interquartile range) for skewed distributions (^a^). The independent t-test (or Mann-Whitney U test for non-parametric distributions) and Χ^2^ tests were used to compare characteristics between patients. As a full echocardiography exam including LA size was not available in all patients, the analysis was reduced to 583 patients down from 1485 patients.

BMI, body mass index; eGFR, estimated glomerular filtration rate; TIA, transient ischemic attack; ANGPT2, angiopoietin 2; BMP10, bone morphogenetic protein 10; CRP, high-sensitivity C-reactive protein; CA125, cancer antigen 125; ESM1, endothelial cell specific molecule 1; FGF23, fibroblast growth factor 23; FABP3, fatty acid binding protein 3; GDF15, growth differentiation factor 15; IGFBP7, insulin like growth factor binding protein 7; IL6, interleukin 6; NTproBNP, N-terminal pro-B-type natriuretic peptide; TnT, high-sensitivity cardiac troponin T.

**SUPPLEMENTAL FIGURES**


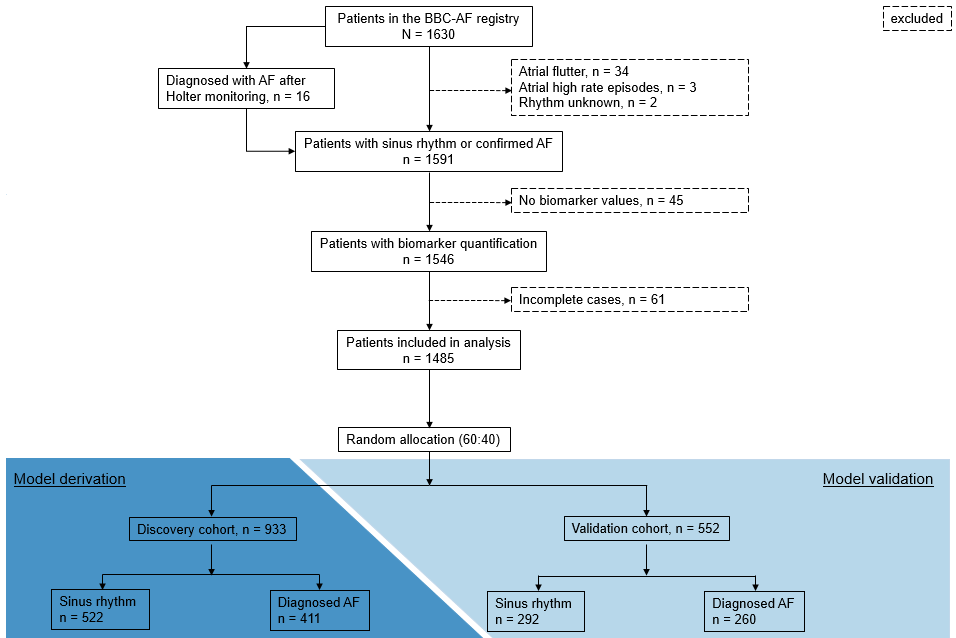


**Fig S1:** **Flowchart of patients included in the analysis.** Patients with arrhythmias other than atrial fibrillation (AF) were excluded. As only complete cases were used, imputation of missing data was not performed. Patients were randomly allocated to the model derivation and validation cohort by a 60:40 ratio.

AF, atrial fibrillation.


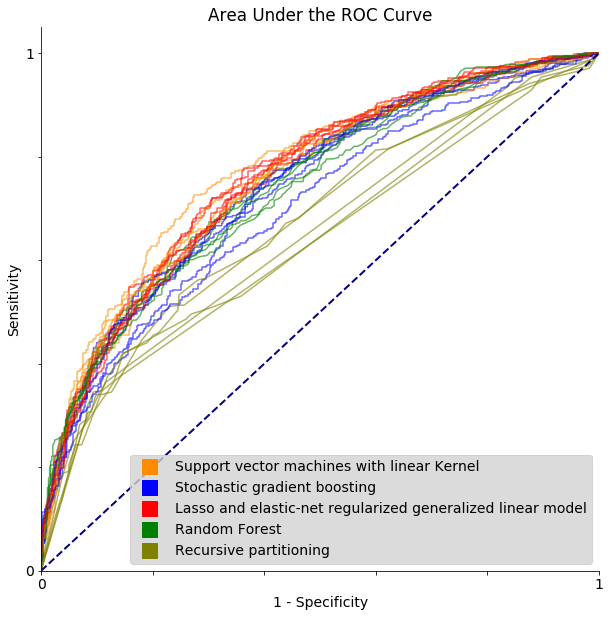


**Fig S2: Comparison of 5 machine learning algorithms (5 iterations per algorithm).** AUC for all developed models and algorithms using data from the validation cohort.

AUC, Area under the ROC curve.


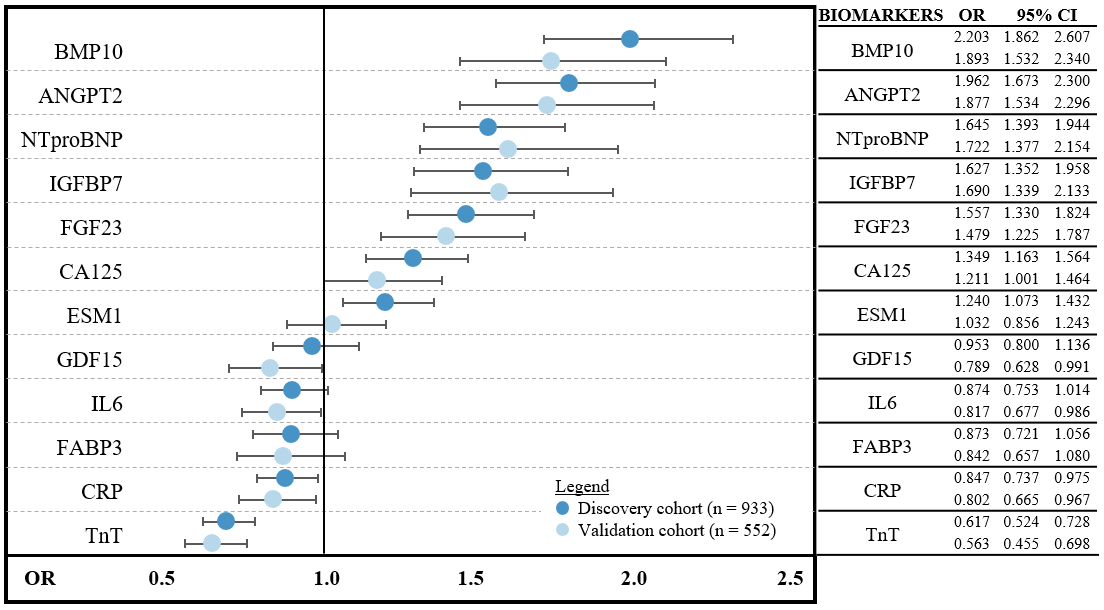


**Fig S3:** **Five biomarkers are predictive of prevalent AF.** Univariate odds ratios (OR) and 95% confidence intervals (CI) of Blom transformed biomarkers (per standard deviation increase) for discovery and validation cohorts also suggest that higher levels of BMP10, ANGPT2, NTproBNP, IGFBP7 and FGF23 are consistently associated with increased odds of prevalent AF. Biomarkers have been adjusted for age, sex, BMI, eGFR, heart failure, stroke/TIA, and hypertension status.

BMI, body mass index; eGFR, estimated glomerular filtration rate; TIA, transient ischemic attack; ANGPT2, angiopoietin 2; BMP10, bone morphogenetic protein 10; CRP, high-sensitivity C-reactive protein; CA125, cancer antigen 125; ESM1, endothelial cell specific molecule 1; FGF23, fibroblast growth factor 23; FABP3, fatty acid binding protein 3; GDF15, growth differentiation factor 15; IGFBP7, insulin like growth factor binding protein 7; IL6, interleukin 6; NTproBNP, N-terminal pro-B-type natriuretic peptide; TnT, high-sensitivity cardiac troponin T; OR, odds ratio; CI, confidence intervals.


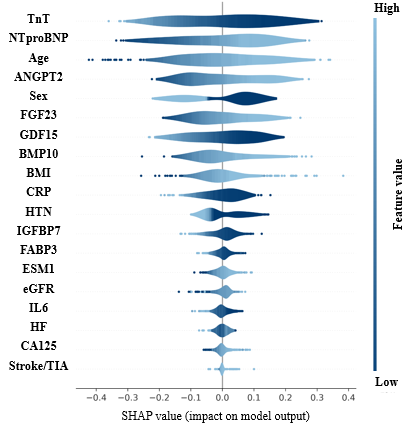


**Fig S4: Visualisation of the SHAP procedure from neural networks analysis using the validation cohort.** Variables are ranked top to bottom from the most to least influential with Blom transformed biomarkers. Dark to light shading indicates low to high variable values. Negative and positive SHAP values correspond to tendencies towards sinus rhythm and atrial fibrillation respectively. High Troponin T being more predictive of sinus rhythm than atrial fibrillation likely attributed to higher proportion of patients in sinus rhythm who have coronary artery disease (n=252/522, 48%) compared to patients with atrial fibrillation (n=93/411, 23%).

BMI, body mass index; eGFR, estimated glomerular filtration rate; TIA, transient ischemic attack; HF, heart failure; HTN, hypertension; ANGPT2, angiopoietin 2; BMP10, bone morphogenetic protein 10; CRP, high-sensitivity C-reactive protein; CA125, cancer antigen 125; ESM1, endothelial cell specific molecule 1; FGF23, fibroblast growth factor 23; FABP3, fatty acid binding protein 3; GDF15, growth differentiation factor 15; IGFBP7, insulin like growth factor binding protein 7; IL6, interleukin 6; NTproBNP, N-terminal pro-B-type natriuretic peptide; TnT, high-sensitivity cardiac troponin T.

**
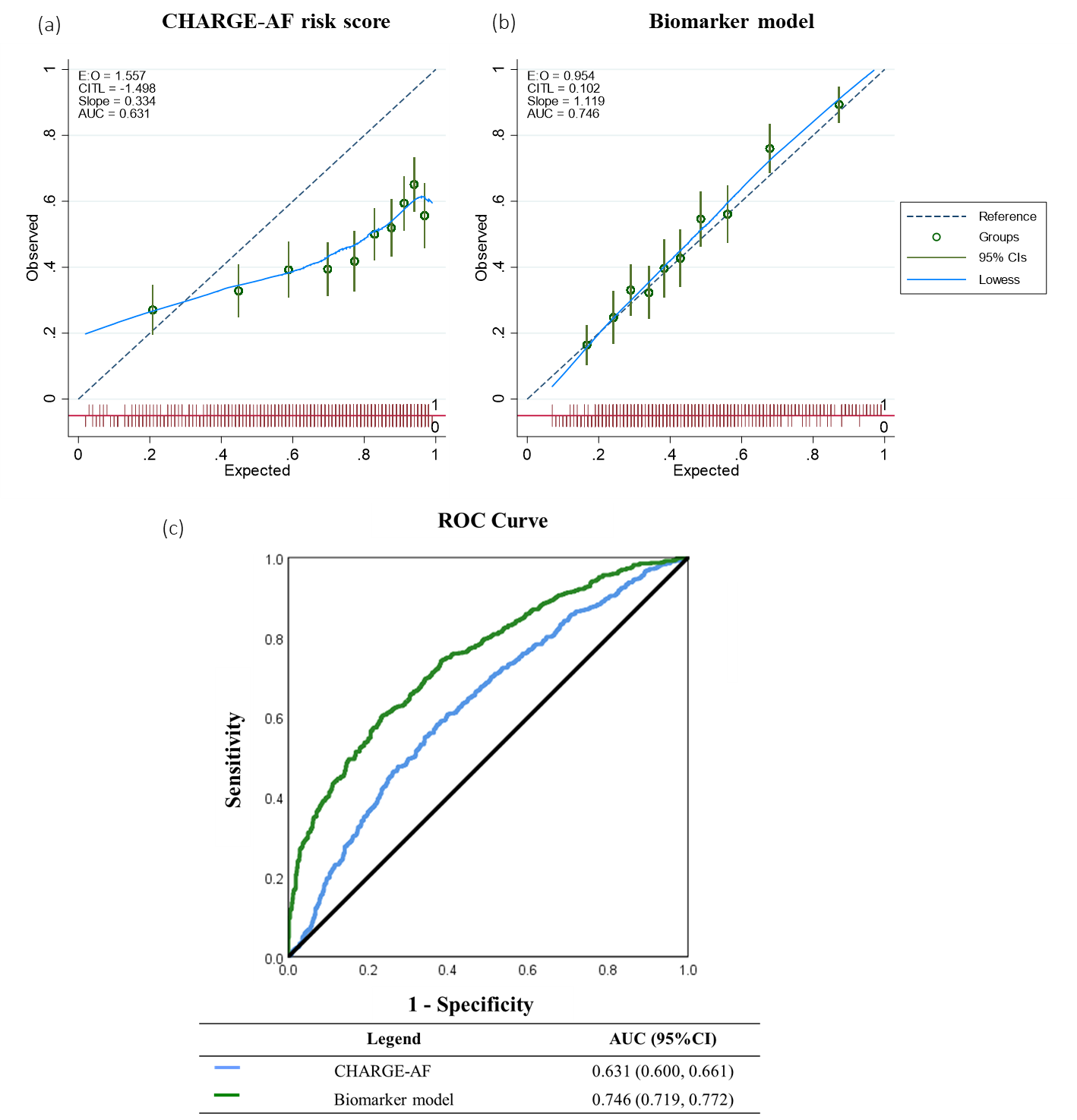
**

**Fig S5: The biomarker model performs better in identifying patients with atrial fibrillation (AF) than the CHARGE-AF score.** Calibration plots comparing the performance of the (a) CHARGE-AF risk score and (b) biomarker model (age, sex, BMI, BMP10, ANGPT2, FGF23) and (c) area under the ROC curves (AUC) with corresponding 95% confidence intervals (n = 1289).

**
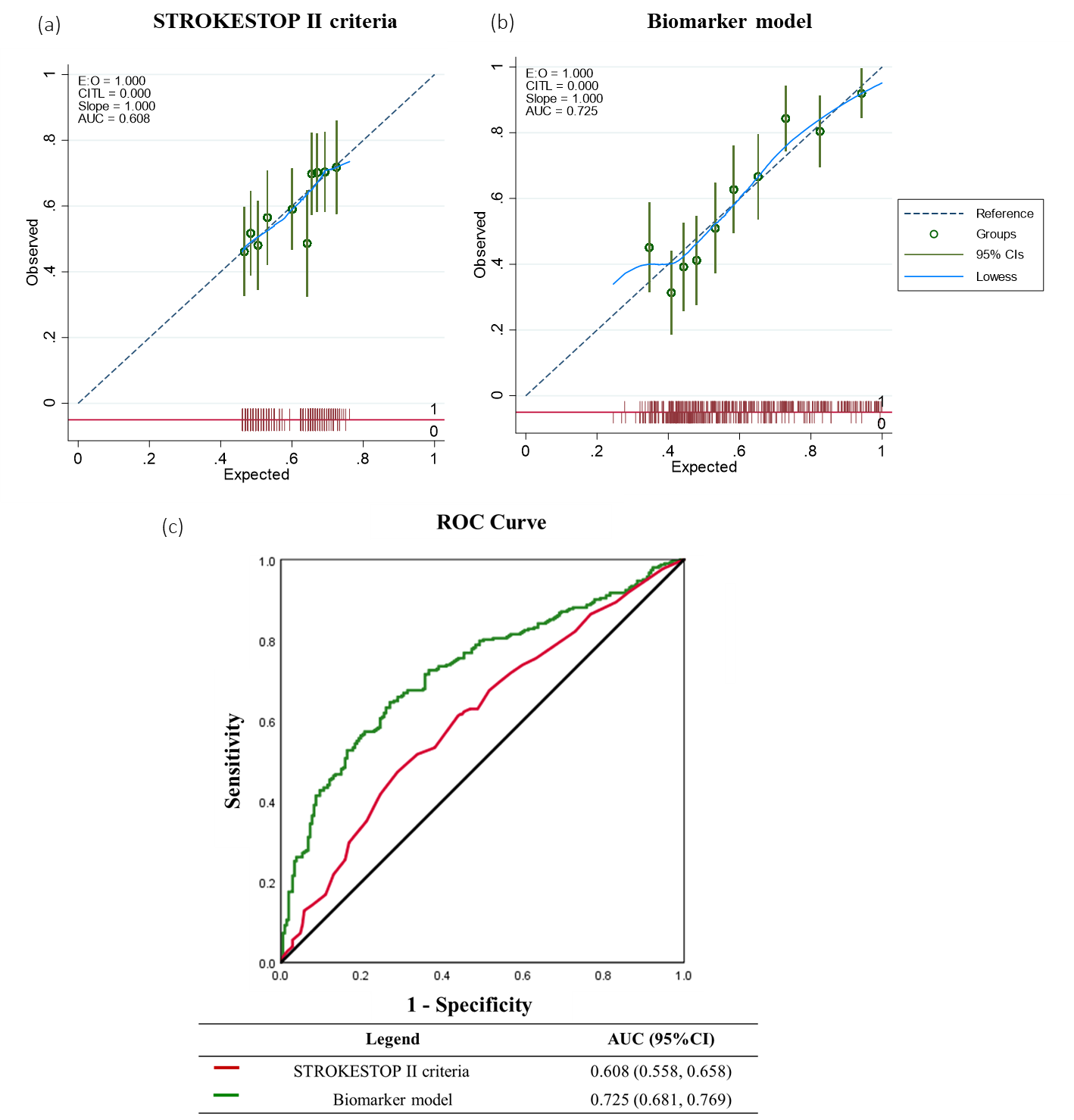
**

**Fig S6: The biomarker model performs better than the STROKESTOP II criteria.** Calibration plots comparing the performance of the(a) STROKESTOP II criteria and (b) biomarker model and (c) area under the ROC curves (AUC) with corresponding 95% confidence intervals (n = 509).

**STROBE_CHECKLIST**

STROBE Statement—Checklist of items that should be included in reports of **cross-sectional studies**

|  | **Item No** | **Recommendation** | **Section** |
| --- | --- | --- | --- |
| **Title and abstract** | 1 | (a) Indicate the study’s design with a commonly used term in the title or the abstract | Abstract |
|  |  | (b) Provide in the abstract an informative and balanced summary of what was done and what was found | Abstract |
| **Introduction** | | | |
| Background/rationale | 2 | Explain the scientific background and rationale for the investigation being reported | Intro paras 1, 2 |
| Objectives | 3 | State specific objectives, including any prespecified hypotheses | Intro para 3 |
| **Methods** | | | |
| Study design | 4 | Present key elements of study design early in the paper | Study population |
| Setting | 5 | Describe the setting, locations, and relevant dates, including periods of recruitment, exposure, follow-up, and data collection | Study population |
| Participants | 6 | (a) Give the eligibility criteria, and the sources and methods of selection of participants | Study population |
| Variables | 7 | Clearly define all outcomes, exposures, predictors, potential confounders, and effect modifiers. Give diagnostic criteria, if applicable | Statistical analysis para 1 |
| Data sources/ measurement | 8* | For each variable of interest, give sources of data and details of methods of assessment (measurement). Describe comparability of assessment methods if there is more than one group | Selection of candidate biomarkers |
| Bias | 9 | Describe any efforts to address potential sources of bias | Biomarker quantification |
| Study size | 10 | Explain how the study size was arrived at |  |
| Quantitative variables | 11 | Explain how quantitative variables were handled in the analyses. If applicable, describe which groupings were chosen and why | Statistical analysis para 1 |
| Statistical methods | 12 | (a) Describe all statistical methods, including those used to control for confounding | Statistical analysis paras 2, 3, Machine learning |
|  |  | (b) Describe any methods used to examine subgroups and interactions | N/A |
|  |  | (c) Explain how missing data were addressed | Statistical analysis para 1 |
|  |  | (d) If applicable, describe analytical methods taking account of sampling strategy | N/A |
|  |  | (e) Describe any sensitivity analyses | N/A |
| **Results** | | | |
| Participants | 13* | (a) Report numbers of individuals at each stage of study—eg numbers potentially eligible, examined for eligibility, confirmed eligible, included in the study, completing follow-up, and analysed | Fig 1 |
|  |  | (b) Give reasons for non-participation at each stage | Fig 1 |
|  |  | (c) Consider use of a flow diagram | Fig 1 |
| Descriptive data | 14* | (a) Give characteristics of study participants (eg demographic, clinical, social) and information on exposures and potential confounders | Participant characteristics, Table 1 |
|  |  | (b) Indicate number of participants with missing data for each variable of interest | N/A (complete case analysis) |
| Outcome data | 15* | Report numbers of outcome events or summary measures | Participant characteristics, Table 1 |
| Main results | 16 | (a) Give unadjusted estimates and, if applicable, confounder-adjusted estimates and their precision (eg, 95% confidence interval). Make clear which confounders were adjusted for and why they were included | Results paras 2, 3 |
|  |  | (b) Report category boundaries when continuous variables were categorized | N/A |
|  |  | (c) If relevant, consider translating estimates of relative risk into absolute risk for a meaningful time period | N/A |
| Other analyses | 17 | Report other analyses done—eg analyses of subgroups and interactions, and sensitivity analyses | Results paras 7, 8 |
| **Discussion** | | | |
| Key results | 18 | Summarise key results with reference to study objectives | Discussion para 1 |
| Limitations | 19 | Discuss limitations of the study, taking into account sources of potential bias or imprecision. Discuss both direction and magnitude of any potential bias | Limitations |
| Interpretation | 20 | Give a cautious overall interpretation of results considering objectives, limitations, multiplicity of analyses, results from similar studies, and other relevant evidence | Implication for stratified therapy |
| Generalisability | 21 | Discuss the generalisability (external validity) of the study results | Clinical implications for identification of AF |
| **Other information** | | | |
| Funding | 22 | Give the source of funding and the role of the funders for the present study and, if applicable, for the original study on which the present article is based | Funding Sources |

*Give information separately for exposed and unexposed groups.

**Note:** An Explanation and Elaboration article discusses each checklist item and gives methodological background and published examples of transparent reporting. The STROBE checklist is best used in conjunction with this article (freely available on the Web sites of PLoS Medicine at http://www.plosmedicine.org/, Annals of Internal Medicine at http://www.annals.org/, and Epidemiology at http://www.epidem.com/). Information on the STROBE Initiative is available at www.strobe-statement.org.
